# Phase Homogeneity and Photothermal Stability in Fully Vacuum-Processed Perovskite Solar Cells

**DOI:** 10.1021/acsenergylett.6c00575

**Published:** 2026-05-05

**Authors:** Isabella Poli, Michele Sessolo, Daniele Meggiolaro, Luca Gregori, Javier Enrique Sebastian Alonso, Maximiliano Senno, Yunseong Choi, Lidón Gil-Escrig, Mirko Prato, Adriana Paracchino, Antonella Treglia, Filippo De Angelis, Henk J Bolink, Annamaria Petrozza

**Affiliations:** † Center for Sustainable Future Technologies, 121451 Istituto Italiano di Tecnologia, via Livorno 60, Torino 10144, Italy; ‡ Instituto de Ciencia Molecular, 16781 Universidad de Valencia, 46980 Paterna, Spain; § Computational Laboratory for Hybrid/Organic Photovoltaics (CLHYO), Istituto CNR di Scienze e Tecnologie Chimiche “Giulio Natta” (CNR-SCITEC), Via Elce di Sotto 8, Perugia 06123, Italy; ∥ Materials Characterization Facility, 121451 Istituto Italiano di Tecnologia, Via Morego 30, Genova, 16163, Italy; ⊥ Centre d’Electronique et de Microtechnique (CSEM), Rue Jaquet-Droz 1, Neuchâtel 2000, Switzerland; # Center for Nano Science and Technology, 121451 Istituto Italiano di Tecnologia, Via R. Rubattino 81, Milan 20134, Italy; ∇ Department of Chemistry, Biology and Biotechnology, University of Perugia, INSTM, Via Elce di Sotto 8, Perugia 06123, Italy; ○ SKKU Institute of Energy Science and Technology (SIEST), Sungkyunkwan University (SKKU), Suwon 16419, Republic of Korea

## Abstract

Vacuum-deposited lead halide perovskite thin films enable solvent-free fabrication, eliminating residual processing solvents that might compromise the long-term stability. Here, we investigate the stability of thermally evaporated mixed-cation compositions FA_0.8_Cs_0.2_PbI_3_ and FA_0.8_MA_0.2_PbI_3_ (FA^+^ = formamidinium and MA^+^ = methylammonium) under thermal and light stress. Although from a thermodynamic perspective the phase stability hierarchy is typically described as MA^+^ < FA^+^ < Cs^+^, with Cs-based perovskites expected to be the most stable, both compositions exhibit thermal robustness, retaining their structural, optical, and morphological properties after continuous heating at 85 °C for over 500 h. Under continuous illumination, however, distinct degradation pathways emerge: FA_0.8_Cs_0.2_PbI_3_ shows the largest morphological and optical changes. This is attributed to chemical inhomogeneities caused by CsI-rich segregations during crystallization, which make point defects effective triggers for photodegradation. Film homogeneity improves by partially replacing iodide with bromide. Based on these results, we selected FA_0.8_MA_0.2_PbI_3_ and FA_0.8_Cs_0.2_Pb­(I_0.8_Br_0.2_)_3_ for device fabrication and evaluated their operational stability. The resulting perovskite solar cells maintain their performance after four months of outdoor operation and withstand 900 h under continuous sun-equivalent indoor illumination at room temperature. These results demonstrate how a high-quality crystallization process can reveal the potential of MA-containing perovskite formulations for long-lived perovskite photovoltaics.

## Graphical abstract

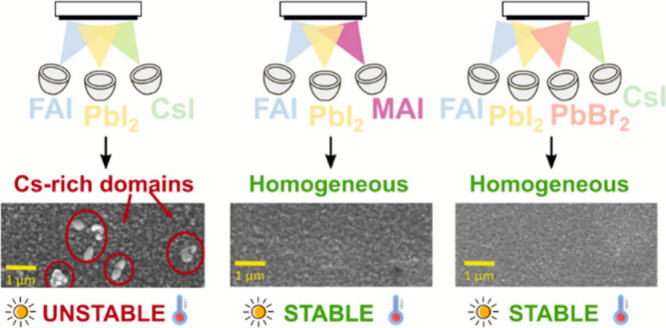

Metal halide perovskites have emerged as promising materials for optoelectronics, enabling the fabrication of high-efficiency and cost-effective photovoltaic devices. One of their key advantages lies in the chemical flexibility of the perovskite structure, which can be tuned through compositional engineering to optimize the optoelectronic performance and stability. In the field of photovoltaics, compositions based on FA_1–*x*_Cs_*x*_PbI_3_ and FA_1–*x*–*y*_Cs_*x*_MA_*y*_PbI_3_ (MA^+^ = methylammonium and FA^+^ = formamidinium) have emerged as some of the most efficient systems.^1–5^ The incorporation of multiple A-site cations has been demonstrated to be essential to improve the structural stability of these materials. In fact, both FAPbI_3_ and CsPbI_3_ tend to adopt nonperovskite yellow phases at room temperature,^6–8^ yet these phases can be suppressed through multication substitution, which relaxes the structural strain and stabilizes the black, semiconducting phase.

The intrinsic phase stability of these materials is further challenged under prolonged heat and light exposure, two stressors that are intrinsic to real-world solar cell operation. A prevailing view in the field is that the inorganic cation Cs^+^ enhances the thermal robustness of perovskites, whereas organic cations, especially MA^+^, tend to accelerate degradation at elevated temperatures.^9–11^ From a thermodynamic perspective, the stability hierarchy is typically described as MA^+^ < FA^+^ < Cs^+^, with Cs-based perovskites expected to be the most stable.^12^ However, recent studies have begun to challenge this assumption. Zhao et al. demonstrated that while Cs^+^ can indeed stabilize perovskite films at high aging temperatures (>100 °C), its presence may be detrimental under milder aging thermal conditions (<100 °C), where MA^+^ instead appears to enhance stability.^13^ This behavior has been attributed to the effect of the perovskite composition on the modulation of the desorption energy barriers, with Cs^+^ incorporation lowering the activation energy for degradation. Furthermore, Li et al. recently showed that conventional FA_1–*x*_Cs_*x*_PbI_3_ films exhibit performance losses due to Cs^+^ accumulation at the surface of the film, causing a valence band offset that increases interfacial carrier recombination.^14^ Notably, this accumulation is not triggered by heat or light but originates during solution-based processing. Similarly, Hidalgo et al. found that Cs-containing compositions were prone to the formation of nonperovskite secondary phases, an effect that could be mitigated through halide alloying, such as partial substitution of iodide with bromide.^15^

To date, most of these insights into perovskite stability have emerged from studies on thin films, whose crystallization is mediated by solvents, a process which has an effect on long-term stability.^13,16–19^ To isolate the role of processing impurities and focus on intrinsic stability, solvent-free, processed thin films represent an ideal case study. In this work, we systematically investigate the intrinsic thermal and photochemical stability of a series of thermally evaporated lead halide perovskite films under an inert atmosphere. Using FAPbI_3_ as the base composition, we explore the impact of mixed A-site with Cs^+^ and MA^+^ to form FA_0.8_Cs_0.2_PbI_3_ and FA_0.8_MA_0.2_PbI_3_, respectively. Our results reveal that all compositions exhibit remarkable thermal stability in the dark, remaining structurally and optically unchanged after 500 h at 85 °C. In contrast, simultaneous exposure to heat and light induces significant degradation in Cs-containing samples, while MA-containing films show superior stability under these combined stress conditions. We investigate the role of phase impurity and point defects, and eventually we extend our study to complete solar cell devices based on FA_0.8_MA_0.2_PbI_3_ operating under indoor and outdoor maximum power point (mpp) tracking conditions. We observe lifetimes of 900 h at room temperature (ISOS-L1I) and 200 h under indoor conditions at 85 °C (ISOS-L2I), and we find virtually no degradation of perovskite solar cells after four months of outdoor operation (ISOS-O2).

Thin perovskite films were grown by thermal coevaporation following the methods reported in the Supporting Information (SI). All films are deposited on ITO-coated glass substrates (where ITO stands for indium tin oxide), precoated with TaTm (*N*4,*N*4,*N*4″,*N*4″-tetra­([1,1′-biphenyl]-4-yl)-[1,1′:4′,1″-terphenyl]-4,4″-diamine), a common hole transport layer (HTL) used in vacuum-deposited perovskite solar cells,^20^ to ensure consistent growth conditions and facilitate direct correlation with solar cell performance. To assess the stability of different perovskite compositions, we subjected the films to controlled aging under three distinct conditions: (i) thermal stress at 85 °C in the dark; (ii) light exposure at 1 sun illumination, at 21 °C; and (iii) combined light and heat stress at 1 sun illumination at 85 °C. All accelerated aging tests were performed in N_2_-filled chambers to exclude the influence of ambient conditions (oxygen and moisture). The temperature was monitored and measured with a thermocouple placed underneath the glass substrate, and illumination was provided by LED light sources spanning the 360–960 nm range, calibrated to 100 mW cm^–2^. Unless otherwise specified, aging tests lasted 500 h. To monitor the evolution of the films, we performed, before and after aging, UV–vis absorption spectroscopy, steady-state photoluminescence (PL), and X-ray diffraction (XRD) in order to identify evident changes in phases (UV–vis and XRD) and the opening of carrier loss channels (PL); eventually, scanning electron microscopy (SEM) was used to visualize the thin-film morphology.

We first explored the effect of thermal aging on two mixed-cation lead iodides with the following nominal composition: FA_0.8_MA_0.2_PbI_3_ and FA_0.8_Cs_0.2_PbI_3_. FA_0.8_MA_0.2_PbI_3_ films were obtained by cosublimation of PbI_2_, FAI, and MAI, using a process adapted from a published protocol.^21^ FA_0.8_Cs_0.2_PbI_3_ films were obtained using a published protocol but using pure iodide precursors.^22^ Both compositions show excellent thermal stability when aged for 500 h in the dark at 85 °C. Top-view and cross-sectional SEM images show no significant morphological changes (Figure 1a,b and Figure 1f,g), and optical absorption spectra indicate no bandgap changes upon thermal aging (Figure 1d and Figure 1i). These results confirm that temperature alone does not induce degradation in either composition under inert, dark conditions. We then explored the effect of light soaking. In FA_0.8_MA_0.2_PbI_3_, light exposure induces a noticeable increase in the grain size and heterogeneity (Figure 1c). However, optical absorption and PL spectra remain essentially unchanged after aging, suggesting minimal optoelectronic degradation (Figure 1d,e and Figure S1). XRD patterns show no peak shift upon aging (Figure S2, Supporting Information), with only a minor increase in fwhm (∼6%), while the peak intensity decreases significantly (∼35%). Furthermore, the intensity ratio of the (001) perovskite peak to the PbI_2_ peak remains nearly constant (2.2 in the fresh sample vs 2.1 after aging; see Figure S2), indicating negligible PbI_2_ formation.The intensity loss could be attributed to a reduced crystallinity and to the increased surface morphological inhomogeneity due to aging.^23^ In contrast to FA_0.8_MA_0.2_PbI_3_, the FA_0.8_Cs_0.2_PbI_3_ films exhibit profound morphological changes under continuous illumination (Figure 1h). SEM imaging revealed the development of a highly porous surface morphology. These changes are mirrored in the UV–vis spectra, where a pronounced flattening of the absorption profile is observed (Figure 1i), consistent with increased light scattering and the optical effect due to the formation of voids.^24,25^ The steady-state PL emission peak exhibits a redshift after aging under illumination (Figures 1j and S1b). The position of the PL peak is sensitive to film morphology, trap-state density, and film composition. A redshift in PL emission in mixed FA/Cs perovskites is often associated with lower Cs content.^26^ It can also indicate increased structural disorder, such as higher grain boundary density, elevated trap concentrations, and enhanced nonradiative recombination pathways.^27–29^ In the observed case, light soaking leads to the formation of pinholes and increased surface morphological inhomogeneity, which may be linked to a rise in defect density and contribute to the observed PL redshift. However, we cannot exclude the possibility that these morphological changes also promote Cs segregation, which could further shift the PL peak to longer wavelengths (lower energies).

**Figure 1:**
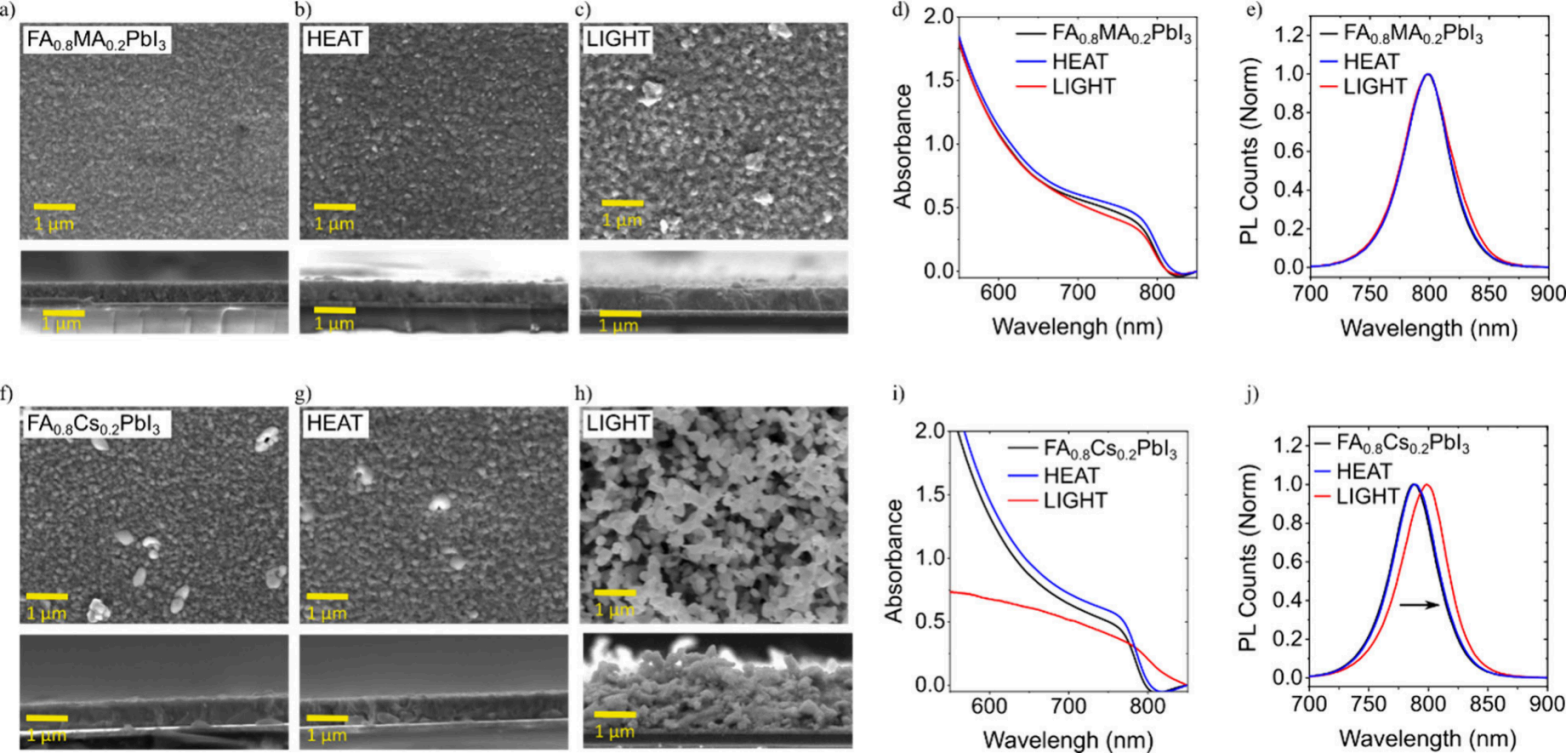
Aging of FA_0.8_MA_0.2_PbI_3_ and FA_0.8_Cs_0.2_PbI_3_ in N_2_ upon heat and light stress for 500 h. (a) Top-view and cross-sectional SEM images of as-prepared FA_0.8_MA_0.2_PbI_3_. Scale bar is 1 μm. (b) Top-view and cross-sectional SEM images of FA_0.8_MA_0.2_PbI_3_ after 500 h at 85 °C in the dark in N_2_. Scale bar is 1 μm. (c) Top-view and cross-sectional SEM images of FA_0.8_MA_0.2_PbI_3_ after 500 h at 21 °C under continuous 1 sun simulated illumination, in N_2_. Scale bar is 1 μm. (d) Optical absorption of as-prepared FA_0.8_MA_0.2_PbI_3_ and after 500 h of aging under 85 °C in the dark and 21 °C under illumination. (e) PL spectra normalized of as-prepared FA_0.8_MA_0.2_PbI_3_ and after 500 h of aging under 85 °C in the dark and 21 °C under illumination (normalized to the maximum intensity). (f) Top-view and SEM images of as-prepared FA_0.8_Cs_0.2_PbI_3_. Scale bar is 1 μm. (g) Top-view and cross-sectional SEM images of FA_0.8_Cs_0.2_PbI_3_ after 500 h at 85 °C in the dark in N_2_. Scale bar is 1 μm. (h) Top-view and cross-sectional SEM images of FA_0.8_Cs_0.2_PbI_3_ after 500 h at 21 °C under continuous 1 sun simulated illumination, in N_2_. Scale bar is 1 μm. (i) Optical absorption of as-prepared FA_0.8_Cs_0.2_PbI_3_ and after 500 h of aging under 85 °C in the dark and 21 °C under illumination. (j) PL spectra normalized of as-prepared FA_0.8_Cs_0.2_PbI_3_ and after 500 h of aging under 85 °C in the dark and 21 °C under illumination.

Then, we evaluated the combined effect of light and heat by aging the films at 85 °C under continuous illumination. As shown in Figure S3, this combined stress significantly accelerates degradation, particularly in FA_0.8_Cs_0.2_PbI_3_. While FA_0.8_Cs_0.2_PbI_3_ films already show substantial degradation after 100 h, FA_0.8_MA_0.2_PbI_3_ remains relatively stable on the same time scale. These results suggest that both materials are sensitive to photodegradation, which is accelerated at elevated temperatures. However, the rate and extent of degradation appear composition-dependent, with Cs-containing compositions degrading faster than those containing MA.

As-prepared FA_0.8_Cs_0.2_PbI_3_ films display surface features of the order of hundreds of nm prior to aging, whereas FA_0.8_MA_0.2_PbI_3_ films are considerably more homogeneous. We performed energy-dispersive X-ray spectroscopy (EDX) line scans across the large surface features in FA_0.8_Cs_0.2_PbI_3_ films, finding them to be enriched in Cs and I, while exhibiting a marked depletion of nitrogen, suggesting a lower concentration of FA^+^ in those regions (Figure S4). These results confirm the presence of Cs^+^-rich domains at the film surface. The presence of unreacted CsI and/or Cs-rich perovskite domains is likely related to its lower reactivity as a consequence of its more ionic bonding. As a result, PbI_2_ is expected to react preferentially with FAI, which can generate more reactive molecular and partially ionized species during evaporation, while Cs incorporation may be less efficient under codeposition conditions. This may lead to compositional inhomogeneities and formation of Cs-rich domains. Notably, this effect is not attributed to differences in sublimation temperature or flux control, as the deposition rates of the individual sources are independently regulated, but rather to kinetic limitations in precursor reactivity during film formation.

To gain further insight into the surface chemistry, we performed X-ray photoelectron spectroscopy (XPS) on FA_0.8_Cs_0.2_PbI_3_ films aged for 100 h at 85 °C under continuous illumination. The films become highly porous, consistent with previous observations (Figure 2a,b). XPS analysis reveals an almost 70% decrease in Cs surface concentration after aging, suggesting Cs migration or diffusion during thermal/photo stress (Figure 2c,d). In contrast, the concentration of the other main elements remains relatively constant. Furthermore, no new chemical states are observed for any of the perovskite-related elements (Figure S5). In pristine films, XRD analysis indicates initially low crystallographic orientation and the presence of secondary phases, including δ-FAPbI_3_ and peaks assignable to CsI (Figure 2e),^30–32^ consistent with surface inhomogeneity and the high Cs content detected by XPS and EDX. Following aging, we observe the disappearance of the δ-FAPbI_3_ peak and a reduced intensity in CsI-related peaks, accompanied by an increase in the perovskite (110) and (003) reflections. This structural evolution suggests partial recrystallization and Cs incorporation into the bulk. The steady-state PL spectrum (Figure 2f) of the aged film also exhibits a blue-shift compared to the as-prepared sample, consistent with a higher incorporation of Cs^+^ within the perovskite structure when compared to the as-prepared FA_0.8_Cs_0.2_PbI_3_.^26^ Finally, we estimated the bulk chemical composition of FA_0.8_Cs_0.2_PbI_3_ films by EDX measurements (Figure S6), finding a clear reduction in the I/Pb ratio after aging at 85 °C under continuous illumination. The Cs redistribution throughout the film with its progressive incorporation into the perovskite lattice is accompanied by a profound morphological transformation, which we attribute to I_2_ expulsion, as evidenced by EDX measurements.

**Figure 2:**
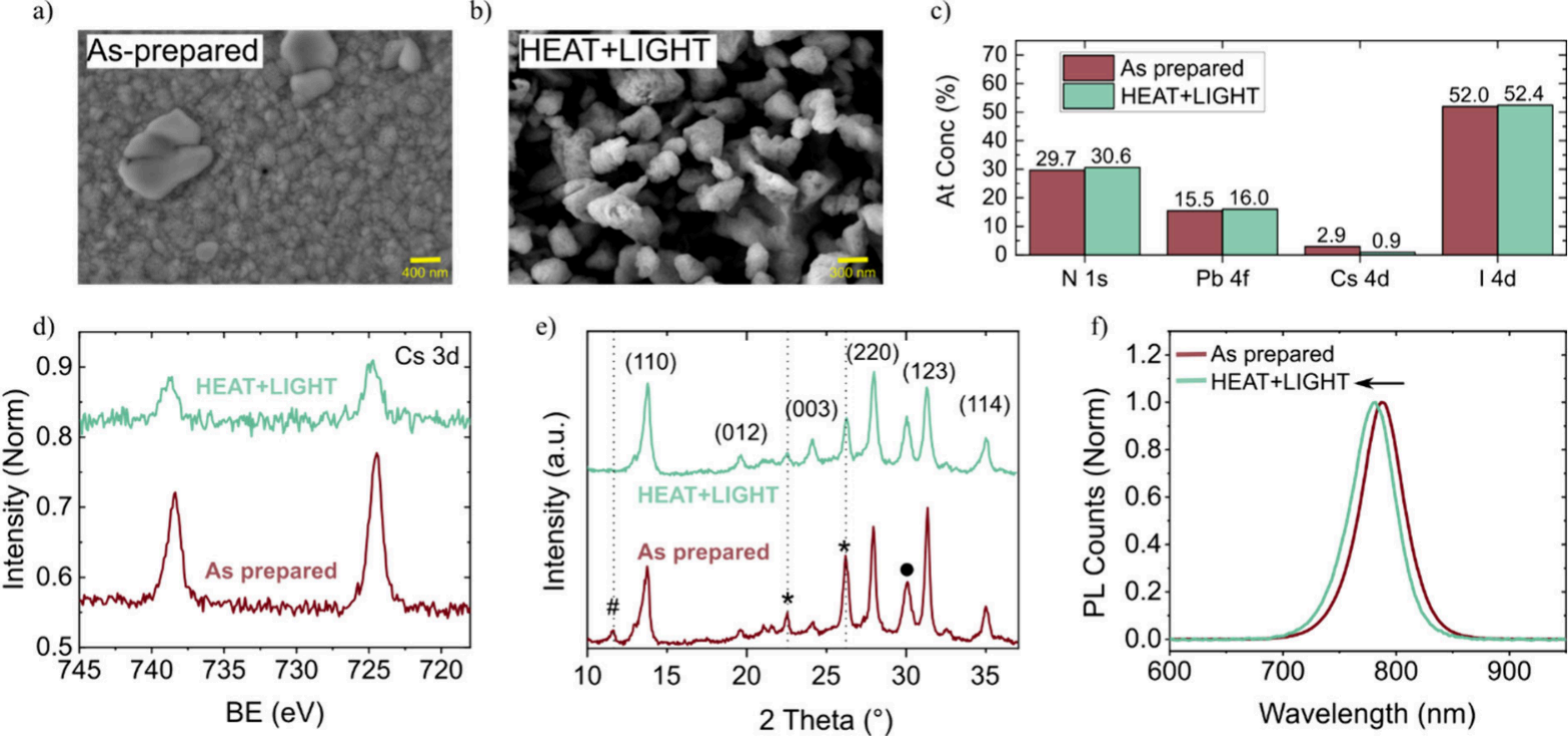
Aging of FA_0.8_Cs_0.2_PbI_3_ in N_2_, under light at 85 °C for 100 h. (a, b) SEM top-view images before and after simultaneous light and heat aging. (c) Atomic concentration taken from XPS of thin films before and after aging. (d) Cs 3d XPS window of the films before and after aging. The spectra have been normalized with respect to the Pb 4f_7/2_. (e) XRD patterns before and after aging. # indicates δ-FAPbI_3_ related peaks. * indicates peaks associated with residual CsI. ● reflects the ITO substrate peak. (f) Steady-state normalized PL spectrum before and after aging (normalized to the maximum intensity).

The results discussed above suggest that combined heat and light exposure promotes redistribution of Cs^+^ ions from surface-enriched regions into the perovskite lattice, facilitating partial recovery of the black perovskite phase. However, this beneficial effect is accompanied by significant morphological degradation. Our SEM and XRD analyses reveal that surface segregation of Cs^+^ leads to the formation of nonphotoactive species such as δ-FAPbI_3_ and unreacted CsI. These findings underscore the critical role of compositional homogeneity in preventing photoinduced degradation in FA_1–*x*_Cs_*x*_PbI_3_ films.^33^ Prompted by this observation, we explored alternative deposition pathways to suppress Cs^+^ segregation. To this end, we introduced partial halide substitution FA_0.8_Cs_0.2_Pb­(I_1–*x*_Br_*x*_)_3_, as it was previously demonstrated that the incorporation of bromine improved the morphology and coverage in solution-processed films.^34,35^ We synthesized three compositions with increasing Br content: *x* = 0.15, 0.2, and 0.27 (EDX quantification in Table S1) and investigated their structural and optoelectronic properties. SEM top-view images show that the incorporation of Br significantly improves the film homogeneity immediately after deposition (Figure 3a–c). Upon light soaking for 500 h at room temperature, these films show markedly enhanced morphological stability, as compared to the pure iodide composition. Specifically, no pinhole formation, voids, or porous structures are observed for *x* = 0.15. With the increment of Br content, voids start appearing, though the structures appear clearly more stable than pure iodide composition. As expected, the addition of Br results in a widening of the bandgap, which is evidenced from the progressive blueshift in both the absorption onset and PL peak with increasing Br content (Figure 3d,e). Then, over 5 min illumination, a redshift of the spectra is observed (Figure 3f), especially in films with *x* = 0.27, accompanied by a significant increase in PL intensity due to the well-known photoinduced halide segregation, which leads to bandgap instability under illumination (Figure S7).^36–38^

**Figure 3:**
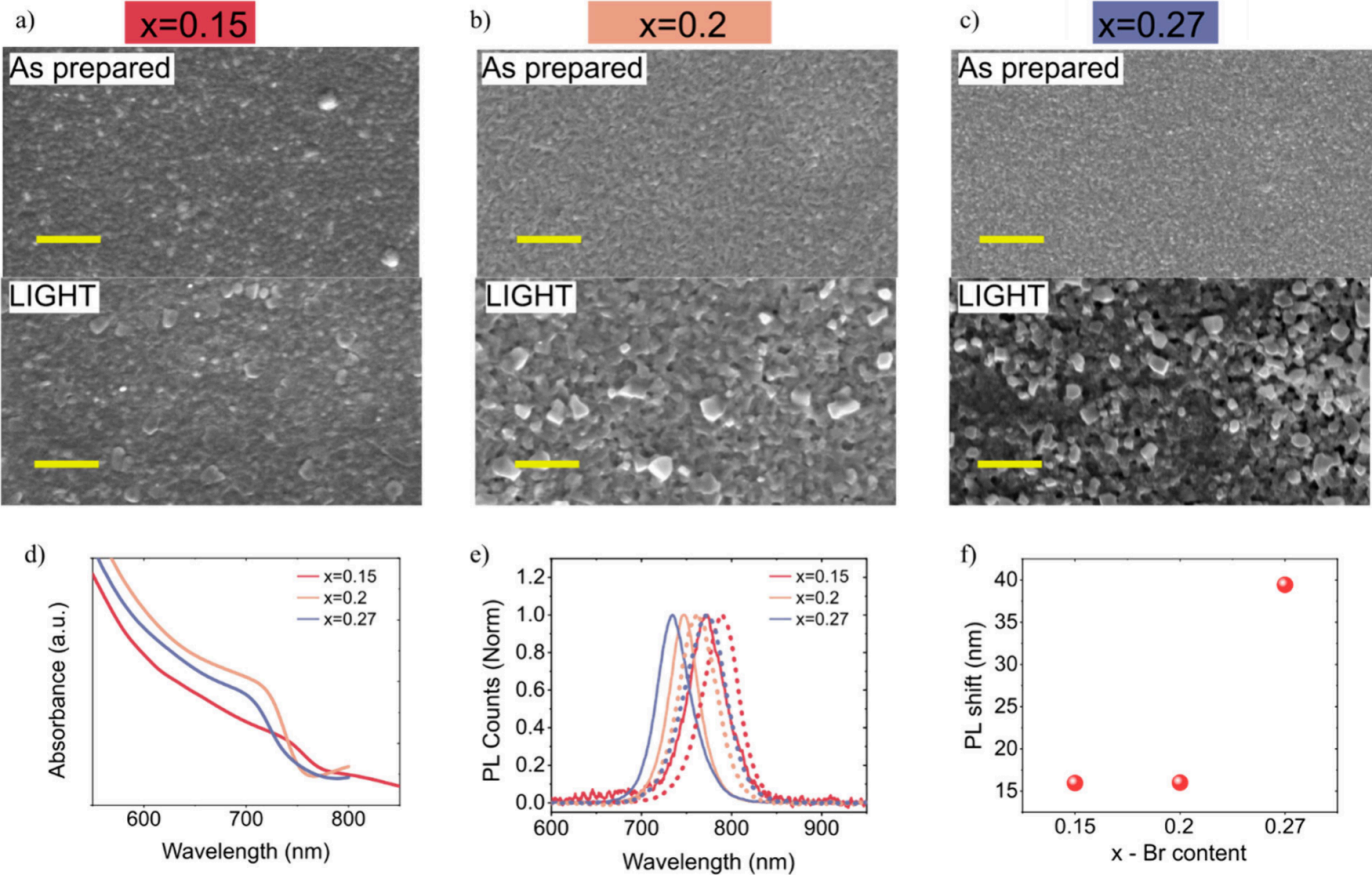
Aging of FA_0.8_Cs_0.2_Pb­(I_1–*x*_Br_*x*_)_3_ in N_2_ with *x* = 0.15, 0.2 and 0.27, under light at room temperature for 500 h. (a–c) Top view SEM images before and after aging of films with different contents of Br. Scale bar is 1 μm. (d) UV–vis of films before aging. (e) Steady-state PL spectra of the films before (solid lines) and after continuous light soaking for 5 min (dotted lines) (normalized to the maximum intensity). (f) PL peak shift after 5 min of continuous illumination.

To rationalize the observed experimental trends, we performed density functional theory (DFT) calculations aimed at studying the lattice stability and the defect chemistry of the perovskites. The effects of chemical composition on the thermodynamic stability of the lattice have been investigated by calculating the heats of formation (Δ_f_ *H*) of the FAPbI_3_ perovskite with 25% of substitutional Cs and MA cations and 33% of substitutional Br in the tetragonal β-phase. Figure 4a shows the optimized unit cells of the perovskite models, while calculated Δ_f_ *H*’s are reported in Table 1.

**Table 1:** DFT-Calculated Heats of Formation (PBE-D3 Level of Theory, Configurational Entropy at 300 K Included) of the Cation- and Halide-Mixed Perovskites in the Tetragonal Phase^a^

Phase	Δ_f_ *H* (meV/f.u.)	DFE@VBM (eV) V_I_^+^/I_i_^–^/I_i_^+^
β-FAPbI_3_	+80	–0.62/1.77/–0.29
β-FA_0.75_MA_0.25_PbI_3_	+72	–0.82/1.65/–0.45
β-FA_0.75_Cs_0.25_PbI_3_	+16	–0.81/1.56/–0.43
β-FAPb(Br_0.33_I_0.66_)_3_	–10	–0.72/2.26/–0.12
β-FA_0.75_MA_0.25_(Br_0.33_I_0.66_)_3_	–28	–
β-FA_0.75_Cs_0.25_Pb(Br_0.33_I_0.66_)_3_	–74	–

a In the last column the calculated defect formation energies of halide defects, i.e., V_I_^+^, I_i_^–^, and I_i_^+^, at the VBM in I-medium conditions are reported (PBE0-D3 level of theory).

**Figure 4:**
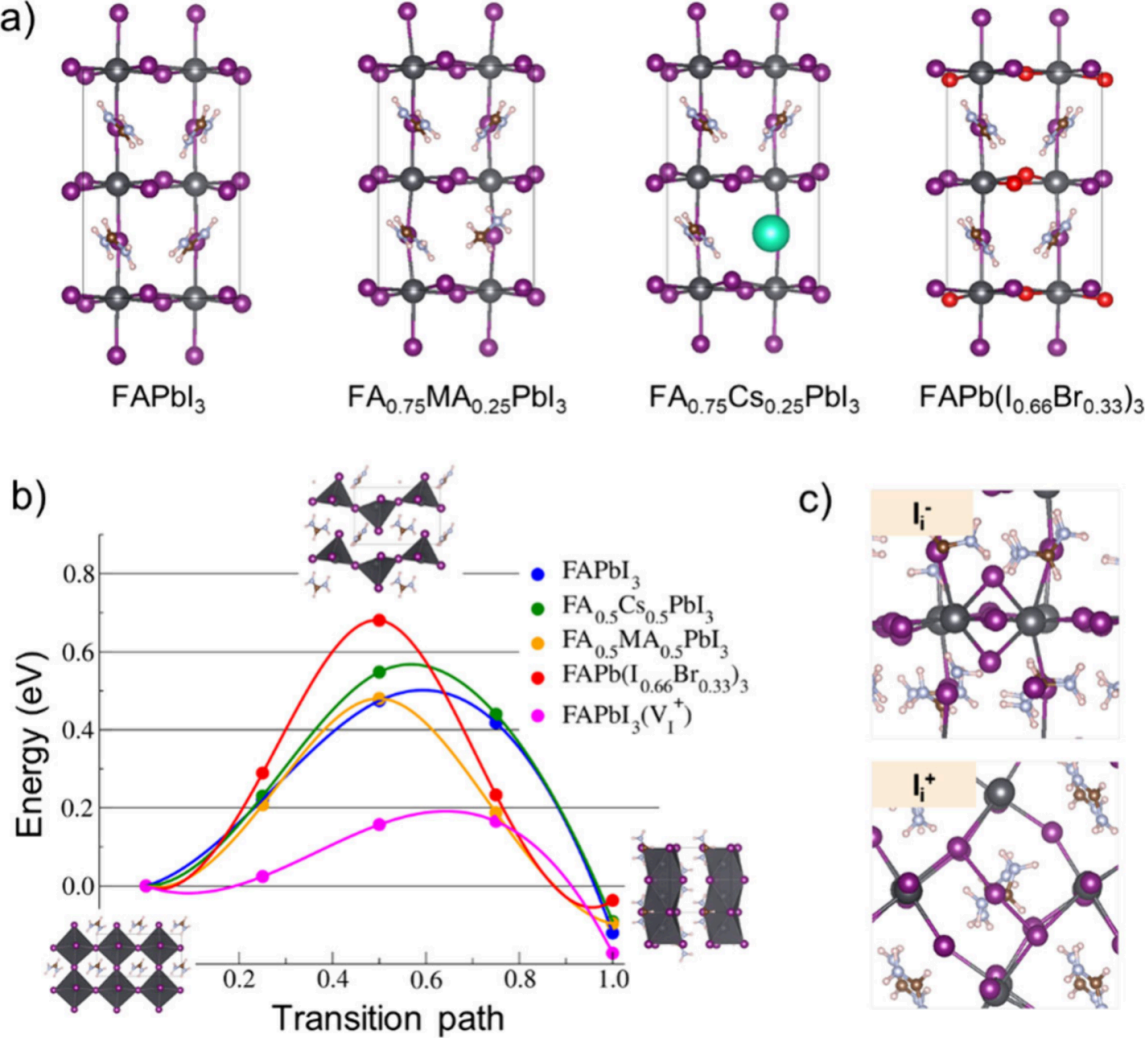
(a) Unit cell structures of the perovskites with different chemical compositions in the tetragonal phase. (b) Energy diagram of the transition between the α and δ phase for the different chemical compositions. To simulate the transition in highly disordered perovskite, one iodine vacancy has been introduced in the cell (magenta line). (c) Equilibrium structures of the I_i_^–^ and I_i_^+^ defects in the β-FAPbI_3_ perovskite.

As reported in Table 1, the mixing of FAPbI_3_ with 25% of Cs^+^ leads to a sensible stabilization of the lattice by 64 meV/formula unit (f.u.), while in the MA^+^ case, a more limited stabilization is observed. The addition of Cs^+^ leads to more thermodynamically stable perovskites compared to MA^+^ as a consequence of the smaller cation radius of Cs^+^ and the reduced tolerance factor.^10^ Mixed-halide compositions with Br^–^ content up to 33% lead to a superior stability, inducing a stabilization of 90 meV/f.u. compared to the pure iodide FAPbI_3_ phase, due to the increase in the ionicity of the lattice. Further stabilization is obtained by tuning the cation site, partially substituting FA^+^ with Cs^+^, with relative stabilization energies comparable to those observed for the FAPbI_3_ phase, while MA^+^ substitution only slightly stabilizes the lattice.

The impact of chemical composition on the relative stability of the perovskite phase vs the photoinactive hexagonal δ-phase has been also analyzed by evaluating the variations in the Δ_f_ *H*’s and the energy barrier of the transition between the α and the δ phase (cell structures in Figure S8). To this aim, linear transit calculations have been performed in the 1 × 1 × 2 supercell of the α phase, carrying 2 f.u. in the cell, and, in the case of the cation-mixed phases, compositions with a (MA^+^ or Cs^+^) to FA^+^ ratio of 1:1 have been simulated to enhance the effects of the cation. The analysis of the calculated Δ_f_ *H*’s highlights that MA, Cs, and Br alloying in the α and δ phases induces lattice stabilization effects comparable with those reported for the β-phase (see Table S2 in SI). Notably, the heats of formation of the δ-phase are in all cases lower than the α and β phases, due to the missing vibrational contributions in the calculation of the Δ_f_ *H*’s which stabilize the perovskite phase above room temperature.^39^

In Figure 4b the energy transition paths for the different chemical compositions are reported. A transition barrier of 0.47 eV/f.u. has been estimated for the pure FAPbI_3_ phase (Figure S9). The mixed cation system with 50% of MA^+^ does not alter the barrier, while a slight increase is reported in the case of 50% of Cs^+^ with an estimated value of 0.55 eV. On the other hand, the mixed-halide perovskite with Br^–^ content up to 33% shows a significant increase of the barrier to 0.68 eV, likely due to the higher stability of the Pb–Br bonds, which are broken in the cubic phase to form the face-sharing configuration in the δ-phase. The potential effects of disorder on the transition have been also investigated by simulating the transition in the presence of an iodine vacancy, as in a highly disordered system. The presence of the iodine vacancy sensibly reduces the barrier to 0.16 eV, leading to a broadening of the energy profile along the transition path. These results show that the partial substitution of FA^+^ with Cs^+^ improves the stability of FAPbI_3_ by inducing a thermodynamic stabilization of the lattice (more negative Δ_f_ *H*) and by slightly increasing the transition barrier toward the δ-phase. Apparently, the partial substitution of FA^+^ with MA^+^ has only very limited stabilization effects on the lattice. Halide alloying of I^–^ with Br^–^ shows larger stabilization effects than cation alloying by inducing both a remarkable stabilization of the lattice and an increase of the energy barrier to the inactive δ phase.

In addition to the thermodynamic stability of the lattice, the robustness of the perovskites under heat and light stress is strictly related to the point defect activity in the material. As previously demonstrated, the photo-oxidation of the lattice with the subsequent I_2_ expulsion at the grain boundaries is a primary photodegradation pathway in lead-halide perovskites.^40^ Such a process is driven by the trapping of the photogenerated holes to form positive interstitials I_i_^+^ and the subsequent annihilation of iodine interstitial pairs (I_i_^+^/I_i_^–^) at the grain surface. The expulsion of iodine induces the formation of vacancies in the lattice, potentially activating the decomposition of the perovskite to the inactive δ phase and leading to the formation of a porous structure. Importantly, we have demonstrated that this holds true both for triiodide and mix-halide lead perovskites.^41^ In the first case one observes photodegradation of the thin film through the expulsion of I_2_;^40^ in the second case, the expulsion of I_2_ is accompanied by destabilization of the bandgap and the formation of iodine rich phases.^42^ To investigate the impact on photostability, we monitored the formation energies of the V_I_^+^, I_i_^–^ and I_i_^+^ halide defects in the FA^+^, MA^+^, and Cs^+^ compositions as well as in the Br-alloyed phase. The formation energies of the V_I_^+^ and I_i_^–^ defects are indicators of the tendency of the material to generate halide Frenkel couples in the lattice and increase ionic disorder. On the other hand, the stability of the I_i_^+^ defect, i.e., the oxidized form of iodide in the bulk material, is indicative of the tendency of the iodide sublattice to be photo-oxidized under light. The calculated DFEs of these defects are reported in Table 1 and Table S3, while the equilibrium structures of the I_i_^–^ and I_i_^+^ defects in FAPbI_3_ are shown in Figure 4c.

DFT results show that the formation energies of halide defects slightly decrease by cation alloying, while in the case of the Br-mixed perovskite, an increase of the formation energies of the I_i_^+^ and I_i_^–^ defects is observed. These results highlight that small fractions of Cs^+^ or MA^+^, up to 25%, do not improve the resistance of the lattice to light stress compared to the pristine phase, while the partial replacement of I with Br induces an increase of photostability.^42,43^ It should be noted that the full replacement of FA cations with MA/Cs to form the pure MA and Cs compositions, beside inducing a larger stabilization of the lattice, also increases the photostability of the perovskite, that strictly follows the thermodynamic stability of the lattice, with FAPbI_3_ being less photostable than MAPbI_3_ and CsPbI_3_, i.e., FAPbI_3_ < MAPbI_3_ < CsPbI_3_.^42^

The predicted theoretical trend seems inconsistent with experimental observations, where FA_0.8_Cs_0.2_PbI_3_ degrades more rapidly under light than does FA_0.8_MA_0.2_PbI_3_. This discrepancy implies that intrinsic thermodynamic stability alone cannot explain the observed degradation behavior. We propose that the enhanced photostability of FA_0.8_MA_0.2_PbI_3_ arises from its superior compositional homogeneity, which mitigates the point defect formation and degradation, while the gain in photostability obtained in mixed halide compositions is offset by the onset of phase segregation under continuous illumination. Notably, there is a complex balance between compositional homogeneity, bandgap stability, and photochemical robustness in halide perovskites. As-prepared FA_0.8_Cs_0.2_PbI_3_ films exhibit compositional inhomogeneity with Cs-rich segregated phases located at the surface. Upon light soaking, these films undergo substantial morphological evolution, becoming increasingly porous. This transformation is attributed to photoinduced I_2_ expulsion, which is accompanied by Cs redistribution throughout the film and its progressive incorporation into the perovskite lattice. In contrast, substitution of Cs with MA (FA_0.8_MA_0.2_PbI_3_) yields significantly more homogeneous films already in the as-prepared state and results in markedly improved morphological photostability, suggesting a reduced photoinduced I_2_ release. To verify that photoinduced I_2_ expulsion is the primary degradation driver, aging experiments were repeated on full device stacks (ITO/TaTm/perovskite/C_60_/SnO_2_/ITO) encapsulated with Al_2_O_3_ to prevent outward diffusion of I_2_. Under these confined conditions, UV–vis spectroscopy and cross-sectional SEM analyses of both FA_0.8_MA_0.2_PbI_3_ and FA_0.8_Cs_0.2_PbI_3_ after 500 h of thermal and combined thermal–light stress reveal negligible optical or morphological changes (Figures S10 and S11), demonstrating that suppressing I_2_ escape dramatically enhances morphological and optical stability.

To assess the technological implications of these compositional effects, we evaluated evaporated perovskite solar cells in a *pin* configuration (glass/ITO/MeO-2PACz/perovskite/C_60_/SnO_2_/Cu/Al_2_O_3_; Figure 5a). We found that despite delivering initial power conversion efficiencies (PCEs) above 18%, FA_0.8_Cs_0.2_PbI_3_-based devices exhibit pronounced instability, with the PCE rapidly decreasing to ∼12% after only 15 min of thermal annealing at 100 °C (Figure S12). Consistent with the material-level observations, we attribute this degradation to structural rearrangements triggered by the intrinsic compositional inhomogeneity of the as-prepared FA_0.8_Cs_0.2_PbI_3_. In contrast, FA_0.8_MA_0.2_PbI_3_-based devices demonstrate significantly enhanced robustness. Based on this superior intrinsic stability, we focused subsequent device optimization efforts on FA_0.8_MA_0.2_PbI_3_-based devices. Current density versus voltage (*J*–*V*) curves for FA_0.8_MA_0.2_PbI_3_ solar cells were recorded under simulated solar illumination, as shown in Figure 5b for a record efficiency device, and the distribution of the corresponding PV parameters is reported in Figure 5c. The highest power conversion efficiency (PCE) obtained is 21.4%, which is among the highest reported for this type of evaporated devices, considering device structure and perovskite type.^44,45^ The average short-circuit current density (*J*_sc_) is 24 mA cm^–2^, with an average open-circuit voltage (*V*_oc_) and fill factor (FF) of 1.09 V and 79.8%, respectively, leading to a PCE of 20.9%. To evaluate the stability of the devices, we tracked their maximum power point tracking (mpp) in inert N_2_ atmosphere, under continuous 1-sun equivalent illumination, at two temperatures: 25 and 85 °C, consistent with the ISOS-L1I and ISOS-L2I protocols (Figure 5d).^46^ Both devices show a linearly decreasing mpp profile over time, which is faster for the samples held at 85 °C. *J*–*V* curves were recorded every 20 min to monitor the evolution of the PV parameters over time during mpp tracking. Figure S13 presents the evolution of each parameter at both temperatures, measured over 1000 h. The devices maintain a rather stable *V*_oc_ throughout the aging test, regardless of temperature. In contrast, the *J*_sc_ was found to decrease over time, particularly for the device aged at 85 °C, where it drops to approximately 50% of its initial value after ∼700 h. The FF initially increases in both cases, reaching a maximum at ∼100 h for the cell aged at 25 °C and at ∼50 h for the cell aged at 85 °C. This increase leads to a transient enhancement in PCE, especially in the sample held at the lower temperature. Such nonlinear early stage behavior has been previously observed and is often attributed to interface effects and charge redistribution at contact layers.^46^ Over extended periods, both devices show a gradual decline in the performance. The T80 lifetime (time to reach 80% of initial PCE) is reached after ∼900 h at 25 °C, while the higher temperature accelerates degradation, reducing T80 to approximately 200 h. As the incorporation of a small amount of Br into the CsFA perovskite leads to significantly improved film homogeneity (Figure 3), we have also prepared analog devices using the FA_0.8_Cs_0.2_Pb­(I_0.8_Br_0.2_)_3_ perovskite formulation and evaluated their operational stability under continuous illumination at 85 °C. As shown in Figure S14, these devices exhibit a stability comparable to that of FAMAPbI_3_-based solar cells (T80 of 220 h), in agreement with the similarly homogeneous morphology observed in the corresponding thin films. This result further supports the link between the material homogeneity and device stability.

**Figure 5:**
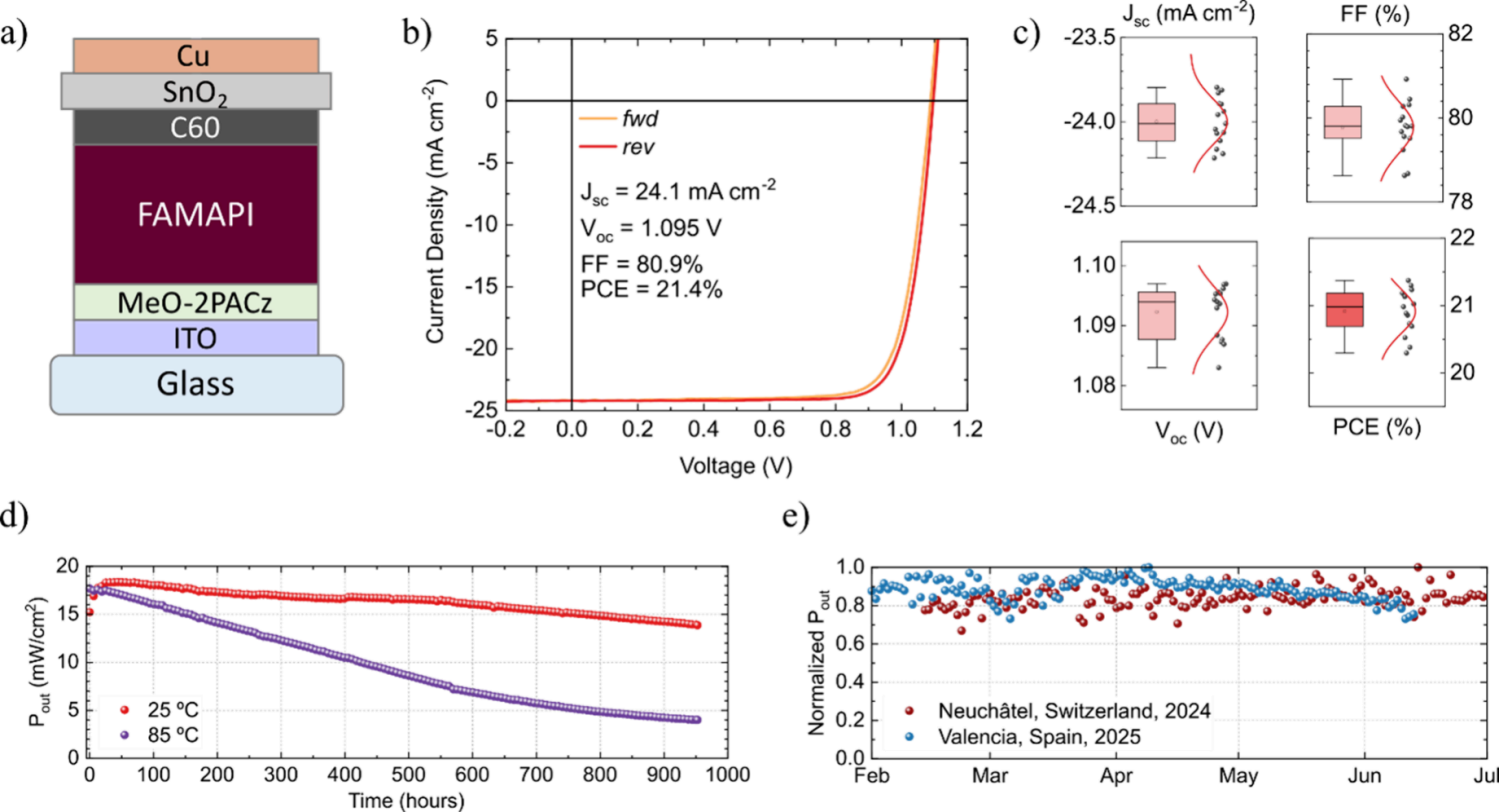
(a) Layout of the perovskite solar cell used in this study. (b) *J*–*V* curves under simulated solar illumination collected in forward (from short to open circuit, orange) and reverse (from open to short circuit, red) bias scan direction. (c) Distribution of the PV parameters obtained from 16 solar cells over different substrates. (d) Indoor MPP tracking of unencapsulated devices at controlled temperature and in nitrogen atmosphere. (e) Outdoor MPP tracking for encapsulated solar cells installed at different geographical locations.

While it is important to assess the relative stability of devices, indoor testing is not sufficient to disclose the real potential of perovskite solar cells, as their metastability is strongly influenced by night and day cycles, among other factors. For this reason, we tested the same device type under outdoor conditions. The samples were encapsulated with a glass–glass lamination process, including edge sealing and ribbon contacts, so that the perovskite cells can be contacted from outside the laminated stack. Samples were tested in two different climate regions, in Neuchâtel (Switzerland, 47° 0′ 0″ N, 6° 56′ 0″ E; climate classification: Cfb, temperate oceanic climate) and in Valencia (Spain, 39° 28′ 12″ N, 0° 22′ 35″ W; climate classification: BSh/Csa, semiarid bordering Mediterranean climate). The mpp as well as the solar irradiance were continuously tracked for several months to estimate the evolution of the device efficiency over time (Figure 5e). Based on data collected over 4–5 months, no degradation is observed in the devices installed in Switzerland. In contrast, a slow but noticeable decline in power output is evident for the solar cells deployed in Spain, during the fourth month outdoor, probably due to the increasing temperatures during spring in Valencia. It is also important to note that the samples were encapsulated differently at the two geographical locations (vacuum lamination vs epoxy lamination in Neuchatel and Valencia, respectively). Hence, at these time scales, we might be observing not only material-related degradation phenomena but likely also the influence of the encapsulation method. Nevertheless, these results are very promising and challenge the commonly accepted concept that MA-containing perovskite formulations are unsuited for applications in view of their low thermal stability. Also, they highlight once again the importance of real-world testing to predict the actual energy yield of perovskite solar cells, due to their unique behavior toward stressors such as light and heat.^47^

To conlcude, we have demonstrated that thermally evaporated FA_0.8_Cs_0.2_PbI_3_ and FA_0.8_MA_0.2_PbI_3_ thin films exhibit remarkable thermal stability, withstanding continuous heating at 85 °C for over 500 h without signs of degradation. In contrast, when the heating is combined with light soaking, optical and morphological degradation becomes evident and particularly pronounced in FA_0.8_Cs_0.2_PbI_3_. We show that Cs tends to segregate during the thin-film formation, which makes the thin film chemically inhomogeneous, boosting the density of point defects which triggers the photo destabilization of the perovskite via iodine expulsion. This picture is corroborated by the improved photostability of the thin film and devices when small amounts of bromide anions are incorporated, thanks to a mitigation of the inhomogeneities as well as a reduced reservoir of potentially oxidizable iodide interstitial defects. Such experimental observations on one side demonstrate that purely organic A-site cations, if high quality crystallization is achieved, can withstand thermal stress, although their phase stability has often been questioned. On the other hand, they call for further investigations into the degree of control of phase-pure metal halide perovskites including Cs cations. While their tendency to segregate was considered a peculiarity of solvent-mediated crystallizations and solubility issues, solvent-free processes were expected to match better the prediction based on a thermodynamic perspective. The latter sees the inclusion of Cs in the perovskite structure as the least expensive from an energetic point of view. However, also through thermal evaporation, achieving chemically homogeneous and phase pure perovskite remains a challenge. To conclude, operational stability tests confirm that FA_0.8_MA_0.2_PbI_3_ devices retain full performance over extended outdoor exposure and prolonged indoor continuous illumination, highlighting their promise for durable perovskite photovoltaics.

## Supplementary Material



## Notes

Experimental section, computational details and additional characterization and discussion, including SEM images, EDX, UV–vis, XRD, XPS fitting parameters, current-density voltage curves and box plots with statistical distribution and variability of key performance PV parameters (PDF)
