# Supplementary material for: Phase Homogeneity and Photothermal Stability in Fully Vacuum-Processed Perovskite Solar Cells
Source: ACS Energy Lett. 2026 May 5;11(6):4437–46. doi: 10.1021/acsenergylett.6c00575 (PMC13270638; doi:10.1021/acsenergylett.6c00575)
Supplement: Supplementary file 1 [file nz6c00575_si_001.pdf]

Supporting Information of

Phase Homogeneity and Photo-thermal Stability in Fully Vacuum Processed Perovskite Solar Cells

Isabella Poli,[1] Michele Sessolo,[2*] Daniele Meggiolaro,[3] Luca Gregori,[3,7] Javier Enrique Sebastian Alonso,[2] Maximiliano Senno,[2] Yunseong Choi,[2] Lidón Gil-Escrig,[2] Mirko Prato,[4] Adriana Paracchino,[5] Antonella Treglia,[6,8] Filippo De Angelis,[3,7,9] Henk J Bolink,[2*] Annamaria Petrozza[6*]

1. *Center for Sustainable Future Technologies, Istituto Italiano di Tecnologia, via Livorno 60, Torino , 10144 ,Italy*
2. *Instituto de Ciencia Molecular, Universidad de Valencia, 46980 Paterna, Spain*
3. *Computational Laboratory for Hybrid/Organic Photovoltaics (CLHYO), Istituto CNR di Scienze e Tecnologie Chimiche "Giulio Natta" (CNR-SCITEC), Via Elce di Sotto 8, Perugia, 06123, Italy*
4. *Materials Characterization Facility, Istituto Italiano di Tecnologia, Via Morego 30, Genova, 16163 Italy*
5. *Centre d'Electronique et de Microtechnique (CSEM), Rue Jaquet-Droz 1, Neuchâtel, 2000 Switzerland*
6. *Center for Nano Science and Technology, Istituto Italiano di Tecnologia, Via R. Rubattino 81, Milan, 20134 Italy*
7. *Department of Chemistry, Biology and Biotechnology, University of Perugia, INSTM, Via Elce di Sotto 8, Perugia, 06123, Italy*
8. *Nanotech@surfaces Laboratory, Empa – Swiss Federal Laboratories for Materials Science and Technology, Dübendorf, 8600, Switzerland*
9. *SKKU Institute of Energy Science and Technology (SIEST), Sungkyunkwan University (SKKU), Suwon 16419, Republic of Korea*

* annamaria.petrozza@iit.it , michele.sessolo@uv.es , henk.bolink@uv.es

Materials and Methods

Materials: lead iodide (99.999%, beads) were purchased from Alfa Aesar; cesium iodide was purchased from Tokyo Chemical Industry (TCI); methylammonium iodide (MAI) and (2-(3,6-Dimethoxy-9H-carbazol-9-yl)ethyl)phosphonic acid (MeO-2PACz) were purchased from Luminescent Technology Corp; formamidinium iodide (FAI) was purchased from Greatcell Solar; tetrakis(dimethylamino)tin(IV) and Trimethylaluminum were purchased from Strem Chemicals.

Evaporated $FA_{0.8}MA_{0.2}PbI_3$*:* The perovskite films were co-deposited from three separate thermal sources containing PbI_2, FAI and MAI, each source having a dedicated quartz crystal microbalance (QCM) sensor. The overall process is controlled with a fourth QCM sensor placed in proximity of the substrate, where the deposition rate at the substrate (r_{SBS}) is monitored. PbI_2 is heated to approximately 280 °C, until r_{SBS} = 0.6 Å/s. Then, FAI is slowly heated and degassed to approximately 140 °C, until a stable reading at r_{SBS} = 0.75 Å/s is achieved. Lastly, the MAI source is heated to approximately 125 °C, until a stable reading at r_{SBS} = 0.80 Å/s. Then the shutter is opened and the deposition carried out until the desired perovskite thickness is achieved.

Evaporated $FA_{0.8}Cs_{0.2}PbI_3$*:* The perovskite films were co-deposited in another vacuum chamber from three thermal sources containing PbI_2, FAI and CsI, each source having a dedicated QCM sensor and shutter. The perovskite was co-deposited using the following approximate parameters: CsI (480−520 °C, 0.1 Å/s), FAI (140−150 °C, 0.45 Å/s) and PbI_2 (270−290 °C, 0.35 Å/s). After stabilization of the different deposition rates, the shutter is opened and the deposition carried out until the desired perovskite thickness is achieved (determined by the reading of a fourth sensor at the substrate).

Solar cells fabrication: glass slides with pre-patterned indium tin oxide (ITO) electrodes were used as substrates. They were subsequently cleaned with soap, water and isopropanol in an ultrasonic bath, followed by 20 min UV-ozone treatment. All the device fabrication is carried out in nitrogen atmosphere without any air exposure. MeO-2PACz was spin coated at 4000 rpm from a 1 mM ethanol solution, followed by annealing at 120 °C for 10 minutes. Perovskite was then deposited as described above. $EDAI_2$ (1 nm) was sublimed on top of the perovskite as interlayer priore to the fullerene deposition. C60 (12 nm) was sublimed in another vacuum chamber. SnOx (20 nm) was deposited on top using previously reported low-temperature atomic layer deposition (ALD) protocols,[1] followed by copper evaporation in a dedicated vacuum chamber. Finally, samples were coated by ALD with 30 nm thick alumina, using another low temperature process.[2] Glass-glass encapsulation in Valencia was carried out by ultrasonically soldering copper cables to the devices, before lamination with a glass slide using a UV-sensitive epoxy resin (Eversolar AB-341). Glass-glass encapsulation in Neuchâtel was done with a vacuum laminator, using contact ribbons and a commercial polyisobutylene edge sealant.

XRD: XRD patterns were recorded with a Bruker D8 Advance diffractometer with Bragg–Brentano geometry equipped with a Cu Kα1 (λ = 1.544060 Å) anode, operating at 40 kV and 40 mA. All the diffraction patterns were collected at room temperature, with a step size of 0.05 in symmetric scan reflection mode, an acquisition time of 1 s.

XPS was carried out on a Kratos Axis UltraDLD spectrometer. The surface of the sample was contacted with the holder through copper tape. Wide scans were acquired at a pass energy of 160 eV, with an energy step of 1 eV, over a (300 x 700) μm^2 area. High-resolution spectra were acquired at a pass energy of 10 eV and energy step of 0.1 eV, over the same analysis area. All

the spectra were obtained using a monochromatic Al Kα source (15 kV, 20 mA). XPS data were analyzed using CasaXPS (version 2.3.24)[3]. To account for possible charging effects, the C 1s peak for adventitious carbon was used as internal reference for the binding energy scale, and its position was set to 288.3 eV.

UV-Vis: absorption spectra were measured on evaporated perovskite thin films using a UV/VIS/NIR spectrophotometer Lambda 1050, PerkinElmer, in the wavelength range 350–850 nm, with step size of 1 nm.

PL: The excitation source was an unfocused beam of a 450 nm c.w. diode laser (Oxxius) or a 525 nm c.w. diode laser (Roithner Lasertechnik GmbH). Photoluminescence was collected in reflection mode at an angle of approximately 45° from the excitation line. Samples were measured in sealed air tight chambers used during the ageing test.

SEM: Both cross section and top view SEM images were obtained using a MIRA3 TESCAN microscope with an accelerating voltage of 5 kV.

EDX: Energy-dispersive X-ray (EDX) analysis was performed using a ZEISS SIGMA 500 Field Emission Scanning Electron Microscope (FESEM) equipped with an Energy Dispersive X-ray (EDX) detector. The instrument was operated at an accelerating voltage of 5 kV for imaging, while an accelerating voltage of 10 kV has been selected for EDX spectra acquisition. A standardless microanalysis that provides quantitative compositional data by evaluating the X-ray spectrum using fundamental physical formulas and general atomic databases was used.

Ageing tests: All accelerated ageing tests were performed in N_2-filled chambers to exclude the influence of ambient humidity. The temperature was kept constant and monitored with a thermocouple placed underneath the glass substrate. Illumination was provided by LED light sources spanning 360-960 nm range, calibrated to 100 mW cm^{-2}.

Device characterization: J-V curves of the solar cells where collected using a Keithley 2612A SourceMeter, using a Wavelab Sinus 70 LED solar simulator and custom-made LabVIEW software. The light intensity was adjusted before every measurement using a calibrated Si reference cell with a KG5 window. The illuminated area was define with a metallic shadow mask (0.1 cm^2, or 0.25 cm^2) placed on top of the device.

Indoor MPPT: Indoor stability tests were performed by means of a P&O Tracker (Arkeo, Cicci Research), under simulated 1 sun illumination (provided by LED lights), in N_2 atmosphere, at MPPT, and at 25 and 85 °C. UV filters were not applied. A JV curve was scanned every 20 minutes to export the main photovoltaic parameters.

Outdoor MPPT: Outdoor stability measurements were performed by means of a MPP Tracker developed by the Laboratory of Photovoltaics and Optoelectronics of the University of Ljubljana,

monitoring at the same time the solar irradiance (with a pyranometer), temperature and humidity, all controlled with custom made software.

(Arkeo, Cicci Research), under simulated 1 sun illumination (provided by LED lights), in N_2 atmosphere, at MPPT, and at 25 and 85 °C. UV filters were not applied. A JV curve was scanned every 20 minutes to export the main photovoltaic parameters.

Computational Details

DFT calculations have been carried with the Quantum Espresso code,[4] by using the Perdew-Burke-Ernzherof (PBE) functional,[5] norm-conserving pseudopotentials,[6] and by including dispersion corrections through the DFT-D3 scheme.[7] The equilibrium structures of the pure and mixed compositions were obtained by optimizing cell parameters and ion positions, by using a cutoff on the wavefunctions of 80 Ry and converged k-point grids in the Brillouin zone (BZ).

Defects have been simulated in the 2x2x2 supercells of the phases. The equilibrium structures of defects were obtained by relaxing ion positions with the PBE functional, by using a cutoff on the wavefunctions of 60 Ry and by sampling the BZ at the Γ point.

The defect formation energies (DFE) have been evaluated by using the expression[8]

$$DFE\ (X^q) = E(X^q) - E(perf) - \sum_i n_i\mu_i + q(E_f + V) + E^q(corr)$$

where E(X^q) is the energy of the supercell containing defect X, E(perf) is the energy of the non-defective system, n and μ are, respectively, the number and the chemical potentials of the iodide species added or subtracted to the non-defective system to form a defect; q is the charge of the defect. Long-range electrostatic interactions Eqcorr have been corrected through the Makov-Payne scheme. I-medium conditions have been simulated by setting the chemical potential of iodine as the average between I-rich (μI = ½ μ(solid I2)) and I-poor conditions (μI = (μ(PbI2)-μ(bulk Pb))/2).

In order to provide more accurate values, the DFEs have been refined by using the hybrid PBE0 functional,[9,10] by performing single point calculations on the PBE optimized structures. Hybrid functional calculations have been performed with the same computational setup used for relaxations and by using a cutoff for the Fock grid of 120 Ry.

Supporting Figures

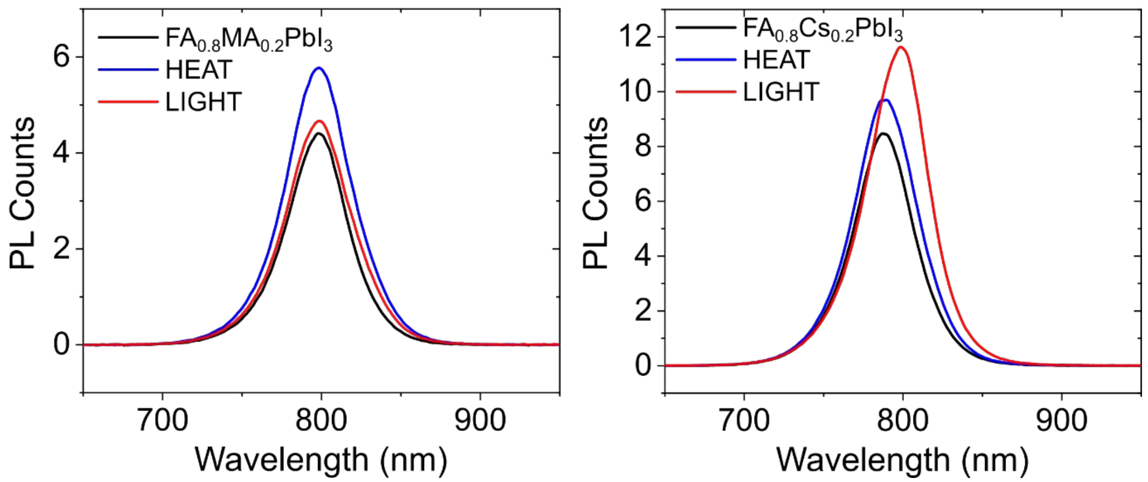

Figure S 1. PL peak of as-prepared $FA_{0.8}MA_{0.2}PbI_3$ and $FA_{0.8}Cs_{0.2}PbI_3$ and after 500 hours of ageing under 85°C in the dark (HEAT) and 21°C under illumination (LIGHT) (excitation source: 525 nm c.w. 2.5 mW, diode laser). Each spectrum has been normalized with respect to the optical density shown in Figure 1d and Figure 1i and the integration time.

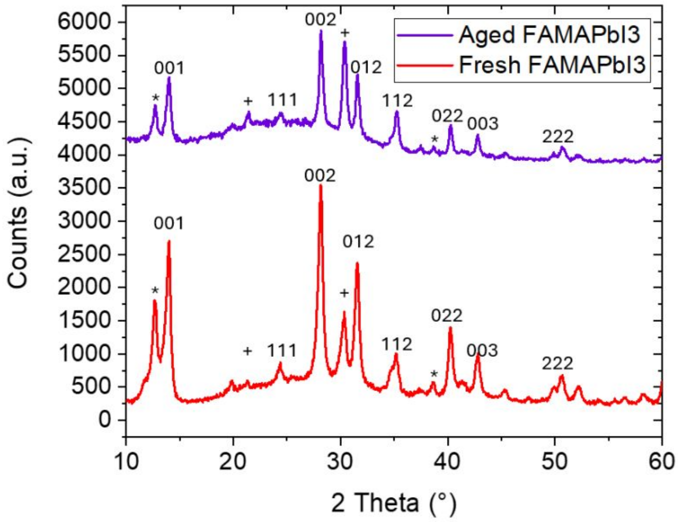

*Figure S 2. XRD patterns of fresh and aged $FA_{0.,8}MA_{0.2}PbI3$ thin films. The material has been aged in N_2, at room temperature under continuous light for 500 hours. * are peaks assigned to PbI_2, while peaks with + symbol are peaks assigned to ITO.*

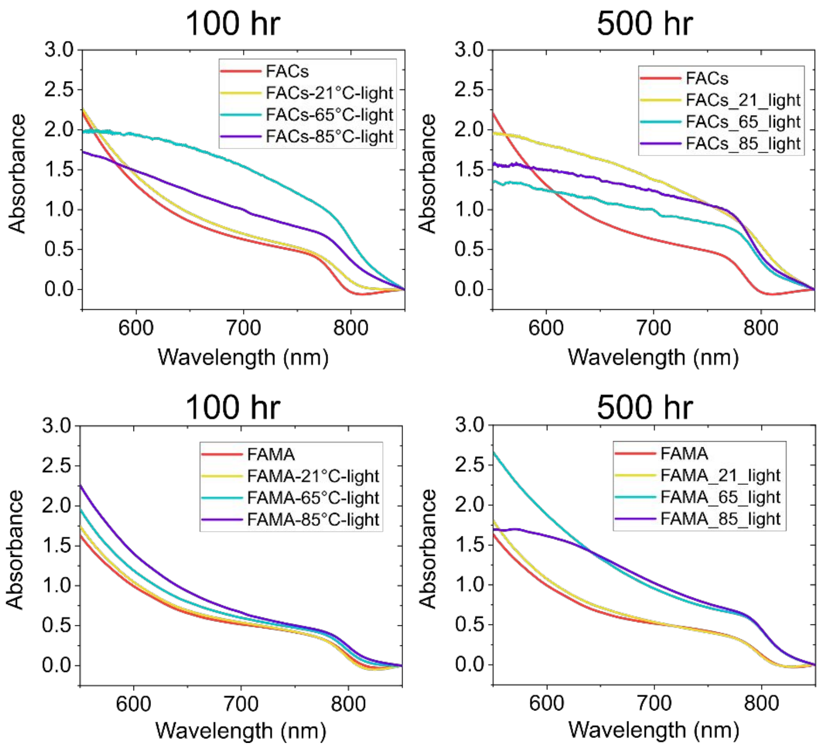

Figure S 3. AGEING of $FA_{0.8}Cs_{0.2}PbI_3$ (top graphs) and $FA_{0.8}MA_{0.2}PbI_3$ (bottom graphs) in N_2, under continuous light at 21, 65 and 85 °C for 100 h and 500 h.

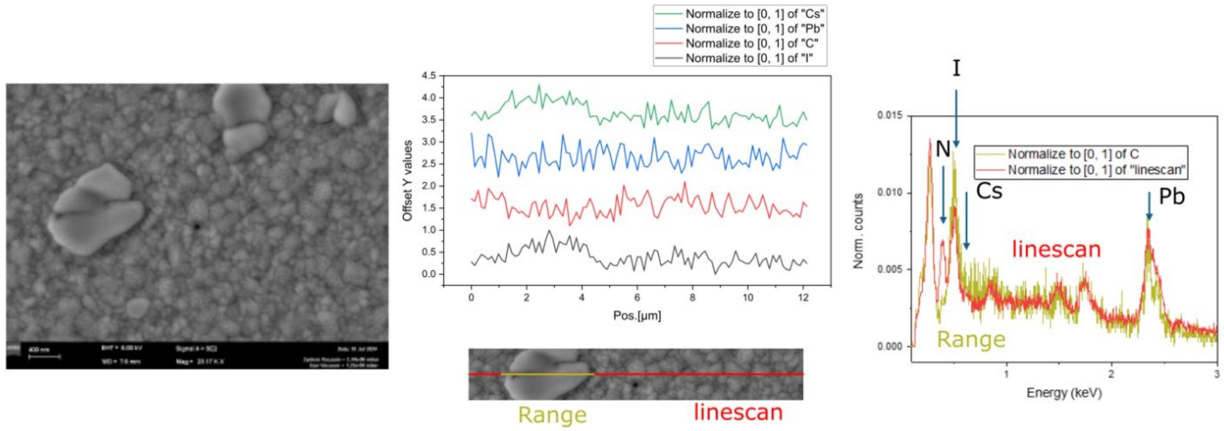

Figure S 4. Top view SEM image of a $FACsPbI_3$ thin film as prepared. Linescans of the EDX integrated signal of the spectral regions of Cs, Pb C, and I. Spectra of the two regions indicated in the inset.

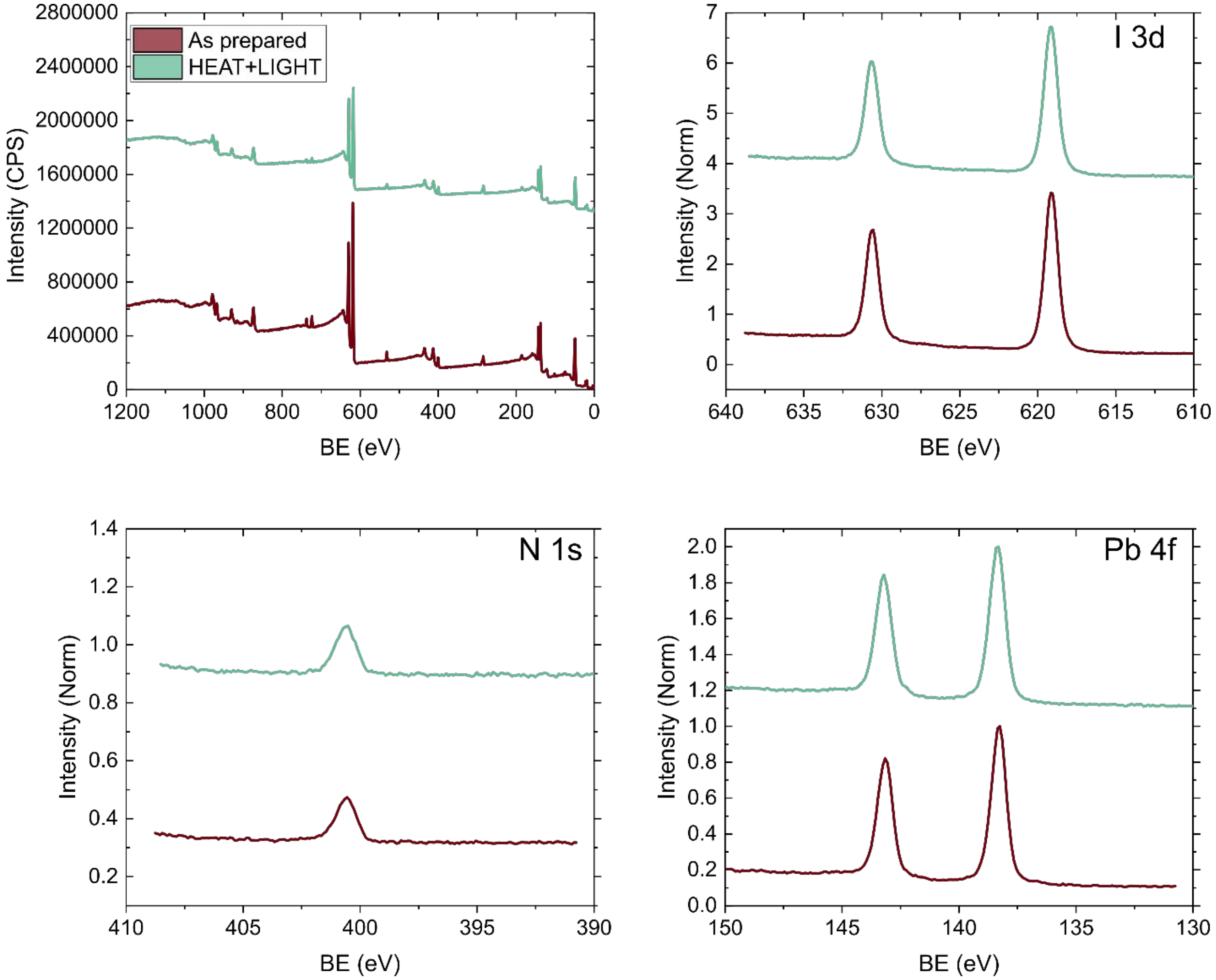

Figure S 5. Wide scan and XPS spectra of the I 3d, N 1s and Pb 4f of $FACsPbI_3$ thin films as prepared and after 100 hours of light soaking at 85°C. XPS spectra of I 3d, N 1s and Pb 4f have been normalised with respect to the Pb $4f_{7/2}$ intensity.

(a) (b)

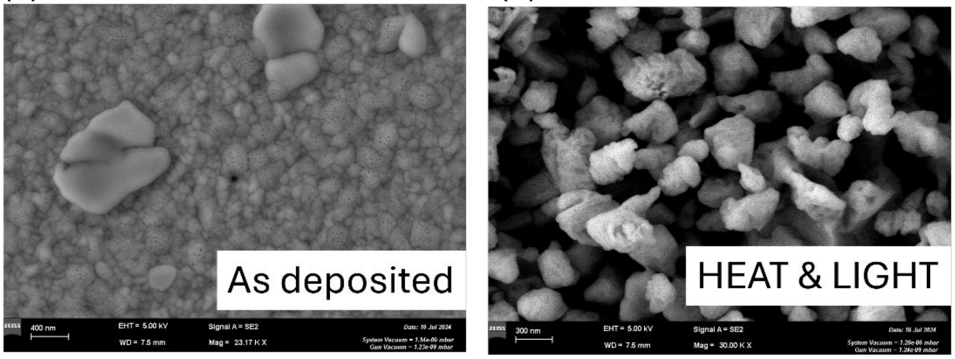

(c)

	I/Pb	
	**As deposited**	**LIGHT & HEAT (100 hr)**
Spot 1	2.90	1.40
Spot 2	1.74	1.71
Spot 3	2.90	1.59
Spot 4	2.08	-
**Average**	**2.41**	**1.57**

Figure S 6. a),b) SEM top view images before and after simultaneous light and heat ageing. (c) Quantitative results of I/Pb ratio obtained via SEM-EDX for $FA_{0.8}Cs_{0.2}PbI_3$ films before and after ageing at 85°C and under continuous illumination.

Table S 1. Quantitative results obtained via SEM-EDX for $FA_{0.8}Cs_{0.2}Pb(I_{1-x}Br_x)_3$ samples with different Br contents. Three different spot areas per sample have been considered.

$FA_{0.8}Cs_{0.2}Pb(I_{1-x}Br_x)_3$		At%			
		Pb	I	Br	Br/I
Low Br	S01	25.759	62.619	9.965	0.16
	S02	25.673	63.631	9.384	0.15
	S03	25.338	62.651	9.865	0.16
Medium Br	S01	25.508	59.024	11.795	0.20
	S02	25.847	58.891	12.282	0.21
	S03	26.153	57.477	11.839	0.21
High Br	S01	24.144	57.046	14.955	0.26
	S02	23.818	57.330	15.279	0.27
	S03	23.791	57.553	15.679	0.27

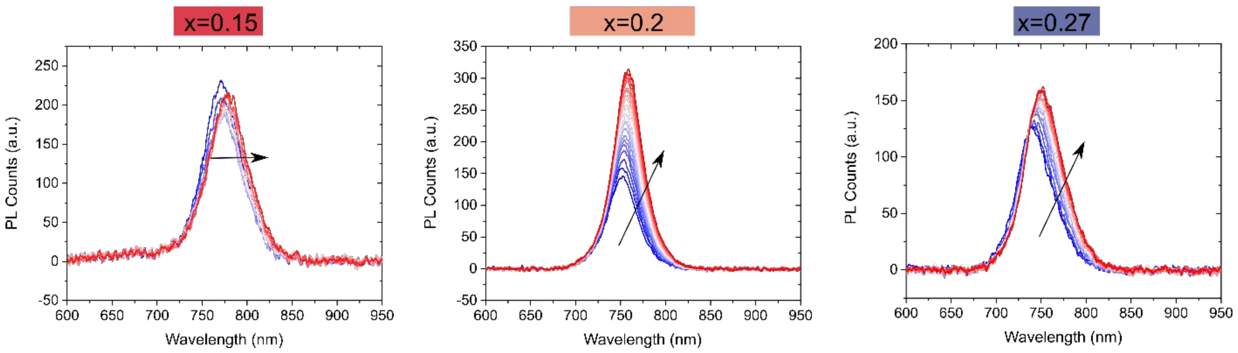

Figure S 7. Evolution of the PL spectra of the mixed halide perovskite samples $FA_{0.8}Cs_{0.2}Pb(I_{1-x}Br_x)_3$ with x=0.15, x=0.2 and x=0.27 as prepared, under continuous illumination with a 525 nm diode laser. PL peaks measured at intervals of 30 seconds for the low-Br content film and 10 seconds for the medium- and high-Br content films.

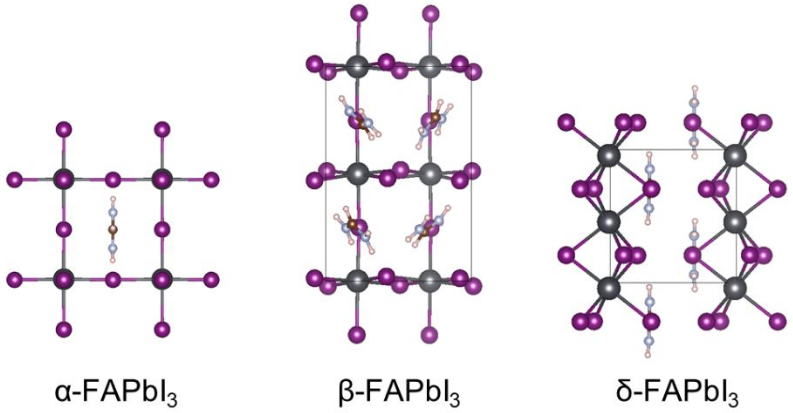

Figure S 8. Cell structures of the α, β and δ phases of the $FAPbI_3$ perovskite.

Table S 2. DFT optimized cell parameters, calculated band gaps and heat of formations ($\Delta_f H$), configurational entropy at 300K included, of the phases studied in this work. All results are calculated at the PBE-D3 level of theory.

**Phase**	**Optimized cell parameters a, b, c (Å) α, β, γ (°)**	**E_g (eV)**	**$\Delta_f H$ (meV/f.u.)**
α-$FAPbI_3$ (1x2x2)	6.39, 12.77, 12.78<br>90.0, 90.0, 90.0	1.64	+146
α-$FA_{0.75}MA_{0.25}PbI_3$ (1x2x2)	6.40, 12.73, 12,74<br>90.0, 90.0, 90.0	1.61	+133
α-$FA_{0.75}Cs_{0.25}PbI_3$ (1x2x2)	6.38, 12.76, 12.76<br>90.0, 90.0, 90.0	1.60	+96
β-$FAPbI_3$ (1x1x1)	8.75, 9.16, 12.82<br>90.0, 90.0, 90.0	1.66	+80

β-$FA_{0.75}MA_{0.25}PbI_3$ (1x1x1)	8.82, 9.13, 12.75<br>90.0, 90.0, 90.0	1.69	+72
β-$FA_{0.75}Cs_{0.25}PbI_3$ (1x1x1)	8.77, 9.10, 12.76<br>90.0, 90.0, 90.0	1.69	+16
β-$FAPb(Br_{0.33}I_{0.66})_3$ (1x1x1)	8.45, 9.01, 12.81<br>90.0, 90.0, 90.0	1.78	-10
β-$FA_{0.75}MA_{0.25}(Br_{0.33}I_{0.66})_3$ (1x1x1)	8.57, 8.86, 12.80<br>90.0, 90.0, 90.0	1.74	-28
β-$FA_{0.75}Cs_{0.25}Pb(Br_{0.33}I_{0.66})_3$ (1x1x1)	8.49, 8.92, 12.76<br>90.0, 90.0, 90.0	1.77	-74
δ-$FAPbI_3$ (1x1x2)	8.59, 8.58, 16.03<br>89.8, 90.1, 119.5	2.79	+27
δ-$FA_{0.75}MA_{0.25}PbI_3$ (1x1x2)	8.60, 8.52, 15.99<br>90.4, 90.4, 119.2	2.75	+9
δ-$FA_{0.75}Cs_{0.25}PbI_3$ (1x1x2)	8.61, 8.57, 15.91<br>89.9, 90.0, 119.8	2.65	-31
δ-$FAPb(Br_{0.33}I_{0.66})_3$ (1x1x2)	8.57, 8.14, 15.94<br>89.9, 90.1, 119.0	2.92	-55

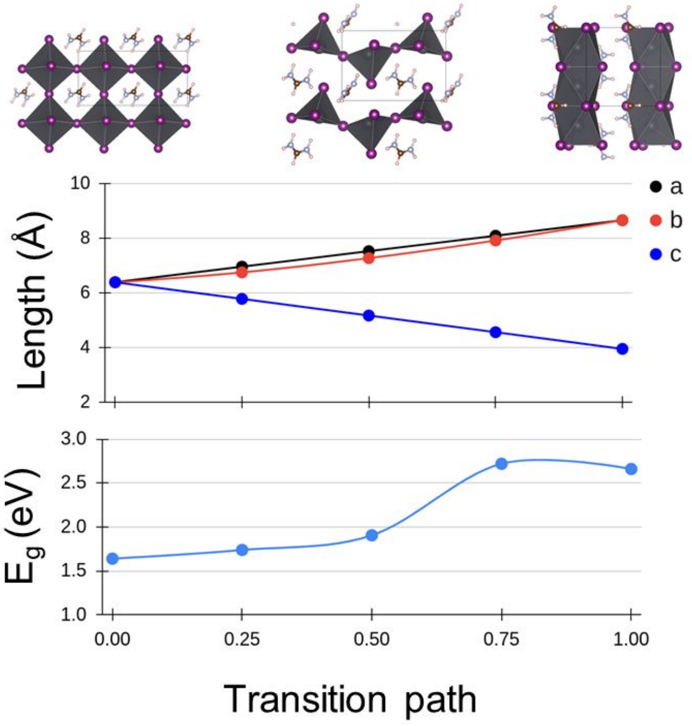

Figure S 9. Evolution of the cell parameters and the band gap along the α to δ phase transition of the $FAPbI_3$ phase.

Figure S8: To estimate the transition barrier between the α and δ phase a linear transit approach has been carried out where ion positions and lattice parameters have been linearly transformed from the cubic to the hexagonal phase and, for each point, ion positions have been relaxed. In Figure S8 the evolution of the lattice parameters and the band gap along the linear transit for the $FAPbI_3$ phase is reported. An expansion of a and b parameters, a contraction of c, and an enlargement of the gamma angle from 90° to 120°, drive the transition. The bandgap increases from 1.64 eV (α phase) to 2.79 eV (δ phase) with a step beyond the central point in the transition path. By following this approach, a thermodynamic barrier of 0.47 eV/f.u. has been estimated for the pure $FAPbI_3$ phase, slightly lower than previous works.[11]

Table S 3. Calculated DFEs at the VBM of halide defects in the 2x2x2 supercells of the cation and halide mixed β-$FAPbI_3$ perovskite (I-medium conditions). Values are calculated at the PBE-D3 level of theory.

**Phase**	**DFE@VBM (eV)**<br>V_I^+ / I_i^- / I_i^+
β-$FAPbI_3$	-0.11 / 1.25 / 0.24
β-$FA_{0.75}MA_{0.25}PbI_3$	-0.19 / 1.10 / 0.19
β-$FA_{0.75}Cs_{0.25}PbI_3$	-0.23 / 1.08 / 0.17
β-$FAPb(Br_{0.33}I_{0.66})_3$	-0.15 / 1.71 / 0.50

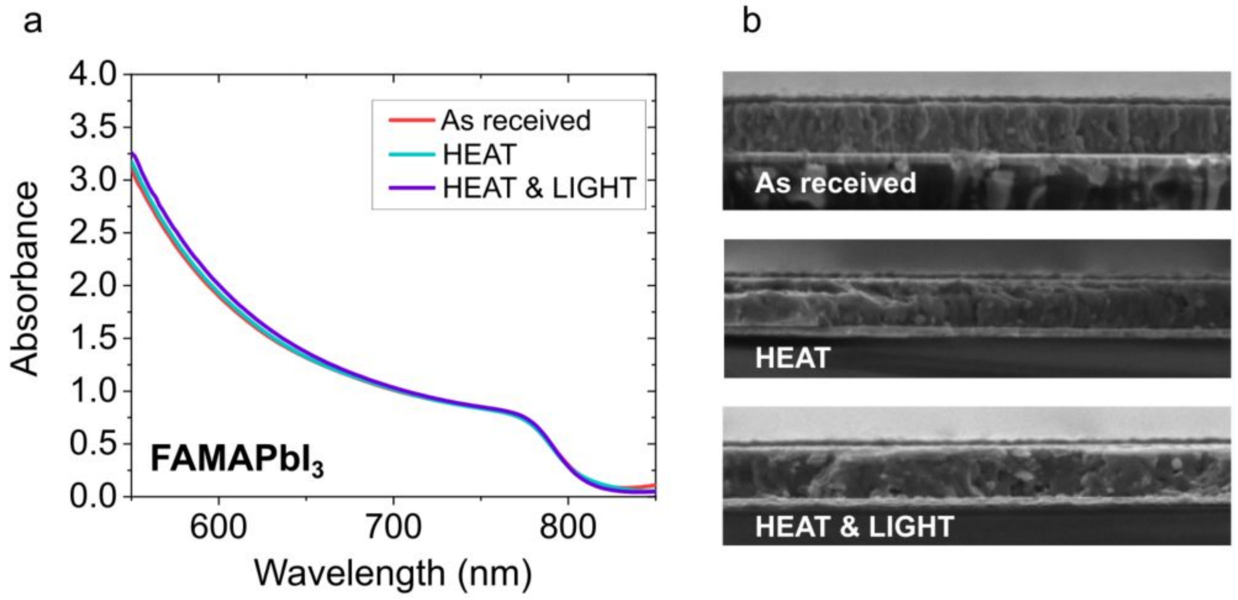

Figure S 10. AGEING of full stack ITO/TaTm/$FA_{0.8}MA_{0.2}PbI_3$/C60/SnO_2/ITO/Al_2O_3. a. UV-Vis of the full stack after 500 h at 85°C in the dark (HEAT) and after 500 h at 85°C and continuous light soaking (HEAT & LIGHT). B. SEM cross section images of the full stack as received and after ageing.

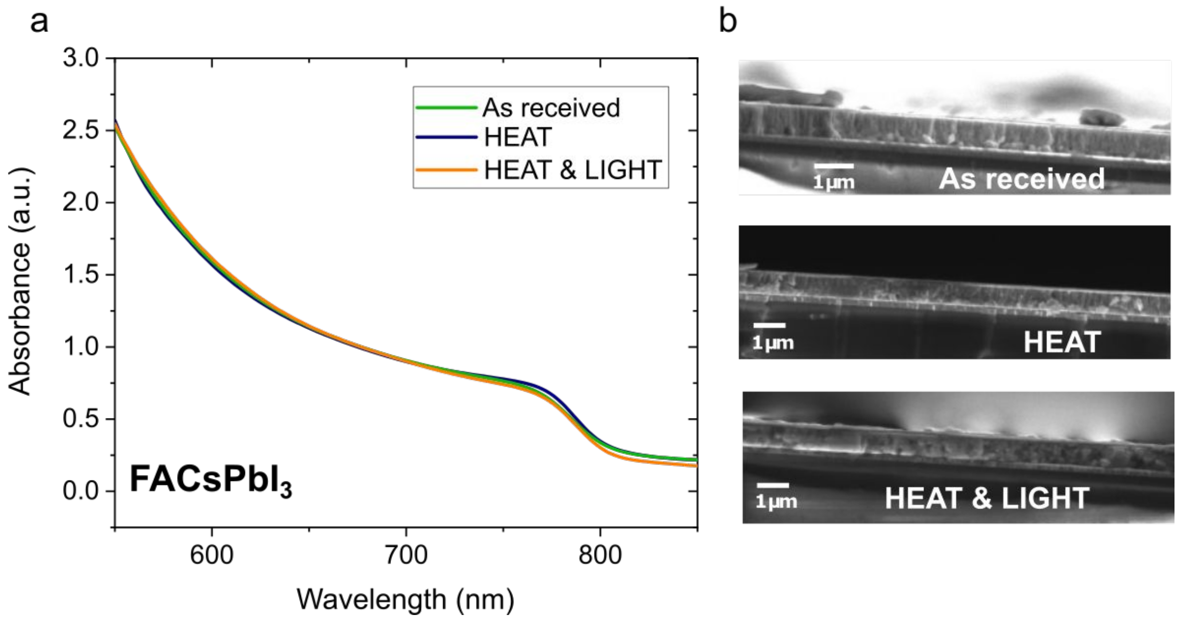

Figure S 11. AGEING of full stack ITO/TaTm/$FA_{0.8}Cs_{0.2}PbI_3$/C60/SnO_2/ITO/Al_2O_3. a. UV-Vis of the full stack after 500 h at 85°C in the dark (HEAT) and after 500 h at 85°C and continuous light soaking (HEAT & LIGHT). B. SEM cross section images of the full stack as received and after ageing.

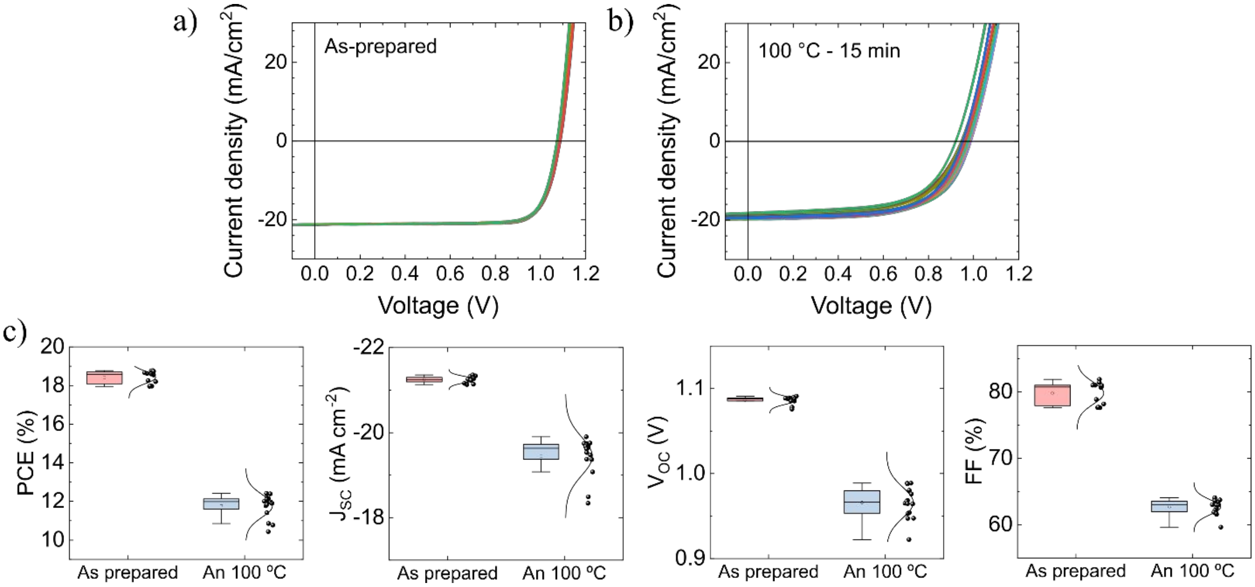

Figure S 12. a) J-V curves of as-prepared $FA_{0.8}Cs_{0.2}PbI_3$-based devices under simulated solar illumination. b) J-V curves of the same $FA_{0.8}Cs_{0.2}PbI_3$-based devices under simulated solar illumination after 15 minutes at 100 °C. (c) Distribution of the PV parameters (PCE, J_{SC}, V_{OC}, FF) of $FA_{0.8}Cs_{0.2}PbI_3$-based devices as-prepared and after 15 minutes at 100°C.

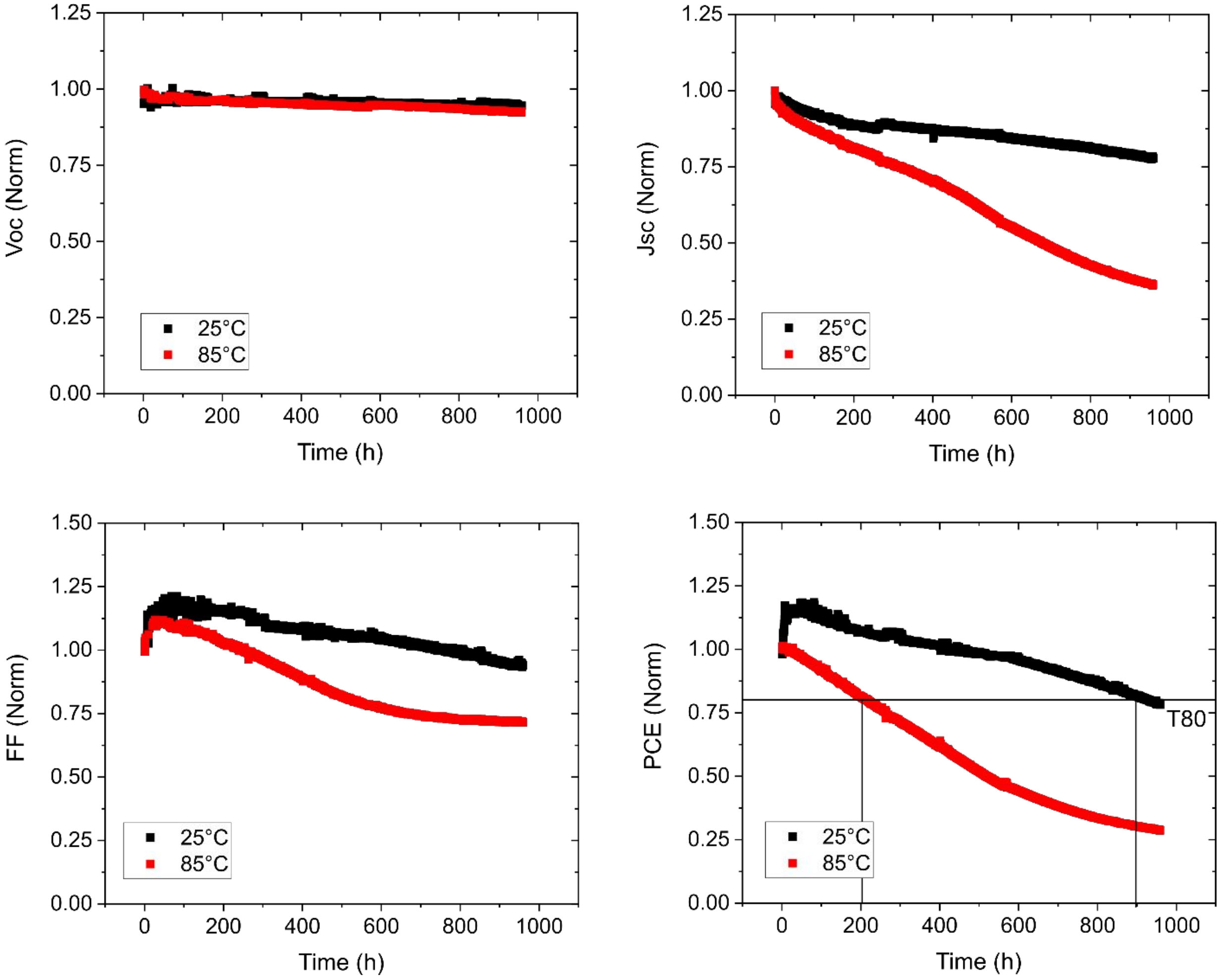

Figure S 13. Individual photovoltaic parameters evolution normalised with respect to the initial value of fully evaporated $FA_{0.8}MA_{0.2}PbI_3$ solar cells measured under mpp, continuous simulated 1-sun illumination, in N_2, at 25 °C and 85 °C. a JV curve was automatically traced every 20 minutes.

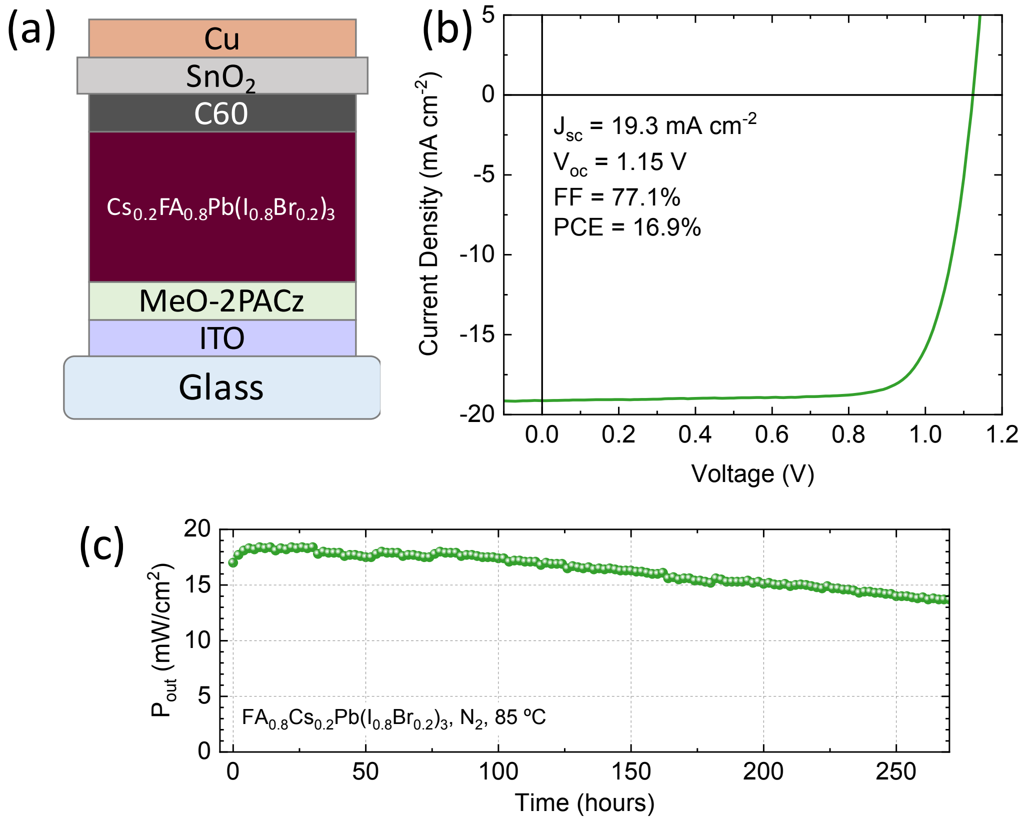

Figure S 14. (a) Schematics of a mixed halide perovskite solar cell and (b) representative J-V curves measured under simulated solar illumination. (c) Maximum power point tracking recorded for encapsulated devices in nitrogen at elevated temperature.

References

1 A. Al-Ashouri, E. Köhnen, B. Li, A. Magomedov, H. Hempel, P. Caprioglio, J. A. Márquez, A. Belen, M. Vilches, E. Kasparavicius, J. A. Smith, N. Phung, D. Menzel, M. Grischek, L. Kegelmann, D. Skroblin, C. Gollwitzer, T. Malinauskas, M. Jošt, G. Matič, B. Rech, R. Schlatmann, M. Topič, L. Korte, A. Abate, B. Stannowski, D. Neher, M. Stolterfoht, T. Unold, V. Getautis and S. Albrecht, *Science (1979).*, 2020, **370**, 13001309.

2 I. C. Kaya, K. P. S. Zanoni, F. Palazon, M. Sessolo, H. Akyildiz, S. Sonmezoglu and H. J. Bolink, *Advanced Energy and Sustainability Research*, 2021, **2**, 2000065.

3 N. Fairley, V. Fernandez, M. Richard-Plouet, C. Guillot-Deudon, J. Walton, E. Smith, D. Flahaut, M. Greiner, M. Biesinger, S. Tougaard, D. Morgan and J. Baltrusaitis, *Applied Surface Science Advances*, 2021, **5**, 100112.

4 P. Giannozzi, S. Baroni, N. Bonini, M. Calandra, R. Car, C. Cavazzoni, D. Ceresoli, G. L. Chiarotti, M. Cococcioni, I. Dabo, A. D. Corso, S. de Gironcoli, S. Fabris, G. Fratesi, R. Gebauer, U. Gerstmann, C. Gougoussis, A. Kokalj, M. Lazzeri, L. Martin-Samos, N. Marzari, F. Mauri, R. Mazzarello, S. Paolini, A. Pasquarello, L. Paulatto, C. Sbraccia, S. Scandolo, G. Sclauzero, A. P. Seitsonen, A. Smogunov, P. Umari and R. M. Wentzcovitch, *Journal of Physics: Condensed Matter*, 2009, **21**, 395502.

5 J. P. Perdew, K. Burke and M. Ernzerhof, *Phys. Rev. Lett.*, 1996, **77**, 3865–3868.

6 M. J. van Setten, M. Giantomassi, E. Bousquet, M. J. Verstraete, D. R. Hamann, X. Gonze and G. M. Rignanese, *Comput. Phys. Commun.*, 2018, **226**, 39–54.

7 S. Grimme, J. Antony, S. Ehrlich and H. Krieg, *Journal of Chemical Physics*, 2010, **132**, 154104.

8 C. Freysoldt, B. Grabowski, T. Hickel, J. Neugebauer, G. Kresse, A. Janotti and C. G. Van De Walle, *Rev. Mod. Phys.*, 2014, **86**, 253–305.

9 J. P. Perdew, M. Ernzerhof and K. Burke, *J. Chem. Phys.*, 1996, **105**, 9982–9985.

10 C. Adamo and V. Barone, *J. Chem. Phys.*, 1999, **110**, 6158–6170.

11 Y. Liang, F. Li, X. Cui, T. Lv, C. Stampfl, S. P. Ringer, X. Yang, J. Huang and R. Zheng, *Nat. Commun.*, 2024, **15**, 1707.
